# Acceptability of a proposed practice pharmacist-led review for opioid-treated patients with persistent pain: A qualitative study to inform intervention development

**DOI:** 10.1177/20494637231221688

**Published:** 2023-12-19

**Authors:** Nicola Cornwall, Charlotte Woodcock, Julie Ashworth, Sarah A Harrisson, Lisa Dikomitis, Simon White, Toby Helliwell, Eleanor Hodgson, Roger Knaggs, Tamar Pincus, Miriam Santer, Christian D Mallen, Clare Jinks, on behalf of the PROMPPT team

**Affiliations:** 1 School of Medicine, 4212 Keele University, Keele, UK; 2 Midlands Partnership University NHS Foundation Trust, Haywood Hospital, Stoke on Trent, UK; 3 Centre for Health Services Studies and Kent and Medway Medical School, 152160 University of Kent, Canterbury, UK; 4 School of Pharmacy and Bioengineering, 4212 Keele University, Keele, Staffordshire, UK; 5 Leek Health Centre, Leek, UK; 6 Division of Pharmacy Practice and Policy, School of Pharmacy, 6123 University of Nottingham, Nottingham, UK; 7 Pain Centre Versus Arthritis, Clinical Sciences Building, City Hospital, Nottingham, UK; 8 UK & Primary Integrated Community Services, Nottingham, UK; 9 Department of Psychology, 7423 University of Southampton, Southampton, UK; 10 Primary Care Research Centre, 7423 University of Southampton, Southampton, UK

**Keywords:** Chronic pain, pain management, acceptability, primary care, opioids, pharmacists

## Abstract

**Introduction:**

Regular review of patients prescribed opioids for persistent non-cancer pain (PCNP) is recommended but not routinely undertaken. The PROMPPT (**P**roactive clinical **R**eview of patients taking **O**pioid **M**edicines long-term for persistent **P**ain led by clinical **P**harmacists in primary care **T**eams) research programme aims to develop and test a pharmacist-led pain review (PROMPPT) to reduce inappropriate opioid use for persistent pain in primary care. This study explored the acceptability of the proposed PROMPPT review to inform early intervention development.

**Methods:**

Interviews (*n* = 15) and an online discussion forum (*n* = 31) with patients prescribed opioids for PCNP and interviews with pharmacists (*n* = 13), explored acceptability of a proposed PROMPPT review. A prototype PROMPPT review was then tested and refined through 3 iterative cycles of in-practice testing (IPT) (*n* = 3 practices, *n* = 3 practice pharmacists, *n* = 13 patients). Drawing on the Theoretical Framework of Acceptability (TFA), a framework was generated (including a priori TFA constructs) allowing for deductive and inductive thematic analysis to identify aspects of prospective and experienced acceptability.

**Results:**

Patients felt uncertain about practice pharmacists delivering the proposed PROMPPT review leading to development of content for the invitation letter for IPT (introducing the pharmacist and outlining the aim of the review). After IPT, patients felt that pharmacists were suited to the role as they were knowledgeable and qualified. Pharmacists felt that the proposed reviews would be challenging. Although challenges were experienced during delivery of PROMPPT reviews, pharmacists found that they became easier to deliver with time, practise and experience. Recommendations for optimisations after IPT included development of the training to include examples of challenging consultations.

**Conclusions:**

Uptake of new healthcare interventions is influenced by perceptions of acceptability. Exploring prospective and experienced acceptability at multiple time points during early intervention development, led to mini-optimisations of the prototype PROMPPT review ahead of a non-randomised feasibility study.

## Introduction

An estimated 43% of UK adults experience persistent non-cancer pain (PNCP),^1^ many of whom are prescribed opioids.^2^ However, evidence for the long-term benefits of opioids is lacking and their use is associated with adverse side-effects and the risk of serious harm, including addiction.^3^ Therefore, regular review is recommended for people prescribed opioids for PNCP to assess treatment effectiveness and, where appropriate, support opioid tapering.^4–6^ However, implementation of best practice guidance is low^7,8^ and routine UK General Practitioner (GP) appointments offer limited opportunity for comprehensive opioid reviews. A move to multidisciplinary working is underway in UK primary care, with more pharmacists working in GP practices.^9,10^ Such practice pharmacists received additional training in patient care and conduct consultations with patients in general practice. Given their skills and knowledge around polypharmacy and complex medicines regimens, practice pharmacists (hereafter pharmacists) seem ideally placed to take a proactive role in reviewing patients prescribed opioids for PCNP, but there is currently no evidence about how they should do this.

**Figure 1.**
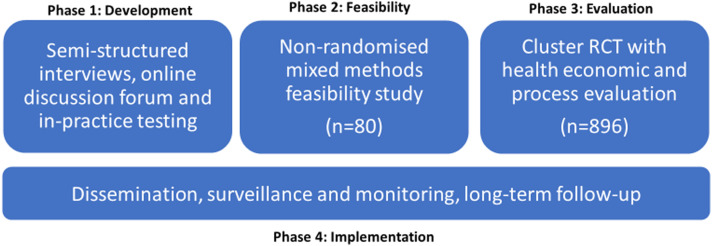
Four MRC phases applied to development and evaluation of the PROMPPT intervention.

The PROMPPT (**P**roactive clinical **R**eview of patients taking **O**pioid **M**edicines long-term for persistent **P**ain led by clinical **P**harmacists in primary care **T**eams) research programme aims to develop and test a pharmacist-led intervention to reduce inappropriate opioid use for persistent pain in primary care (PROMPPT review). The PROMPPT programme is informed by the Medical Research Council (MRC) framework for development and evaluation of complex interventions^11^ (see Figure 1). This framework has four phases: Development, Feasibility, Evaluation and Implementation.

**Figure 2.**
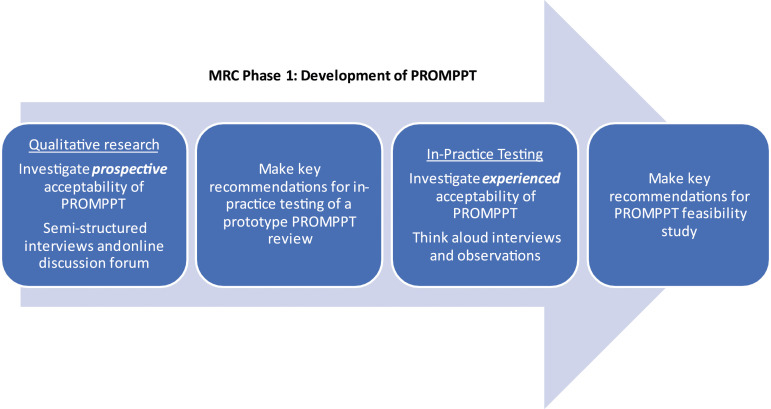
Evaluation of acceptability of PROMPPT.

The current study sits in phase one of the MRC framework and focuses on one aspect of intervention development, developing an intervention that is acceptable to those who will use it. Perceptions of acceptability influence intervention uptake by patients and implementation by health care practitioners. However, until recently the construct of acceptability has been poorly defined and understood.^12,13^ We took a theory-informed approach drawing on the Theoretical Framework of Acceptability (TFA), to explore acceptability of a proposed PROMPPT review (prospective acceptability) and, through in-practice testing, explored acceptability of a prototype PROMPPT review (experienced acceptability). Recommendations for optimising the intervention were made at key timepoints (see Figure 2). Table 1 outlines how we aligned TFA constructs to PROMPPT.

**Table 1.** The theoretical framework of acceptability applied to PROMPPT review development.

TFA constructs	Assessment of acceptability	
	Prospective acceptability of a proposed PROMPPT review in principle	Experienced acceptability of prototype PROMPPT review in practice
Global acceptability	How acceptable will the proposed PROMPPT review be?	How acceptable was the PROMPPT review?
Affective attitude	How do patients and healthcare professionals (HCPs) feel about the proposed PROMPPT review?	What did patients and HCPs feel about the PROMPPT review?
Burden	How easy or difficult will it be to participate in the proposed PROMPPT review?	How easy or difficult was it to participate in the PROMPPT review?
Ethicality	How fair will it be for patients to be offered the proposed PROMPPT review?	How fair was it for patients to be offered the PROMPPT review?
Intervention coherence	How do patients and HCPs think the proposed PROMPPT review will lead to changes in the management of opioids?	How did the PROMPPT review lead to changes in the management of opioids?
Opportunity costs	Will patients and HCPs have to give up things that are important to them to participate in the proposed PROMPPT review?	What did patients and HCPs have to give up to participate in the PROMPPT review?
Perceived effectiveness	Is the proposed PROMPPT review likely to lead to changes in management of opioids?	Did the PROMPPT review lead to changes in management of opioids?
Self-efficacy	How confident would patients and HCPs feel about engaging with the proposed PROMPPT review?	How confident were participants to engage with the PROMPPT review?

## Methods

This study used semi-structured interviews, an online discussion forum and in-practice testing (IPT) with think-aloud interviews, in a UK Primary Care setting (July 2019–February 2020).

### Recruitment and conduct

#### Prospective acceptability

##### Interviews (August 2019–October 2019)

Adult patients (>18 years) prescribed opioids for ≥6 months for PNCP were recruited, by postal invite, from two West Midlands GP practices without a pharmacist. Semi-structured interviews were conducted face-to-face or by telephone, according to participant preference. A topic guide informed by a patient advisory group included; experiences of long-term pain, using regular medication, experiences of discussing pain management with healthcare professionals, knowledge of pharmacists and thoughts about a proposed review with a pharmacist (supplementary material 1). We also included questions related to theoretical constructs of the TFA.

Pharmacists with experience of consultations with patients in UK General Practice were recruited opportunistically from professional networks. Semi-structured interviews were conducted via telephone or face-to-face using a topic guide, informed by the TFA. Questions included experiences of consulting with patients prescribed opioids for PNCP, thoughts about pharmacists delivering a proposed PROMPPT review and possible components of the PROMPPT review (supplementary material 2).

Interviews were conducted by NC, an experienced qualitative researcher, audio-recorded and recruitment stopped when the interviewer (NC) deemed data saturation had been reached whereby nothing new was being heard from the interviewee responses.^14^

##### Online discussion forum (October–December 2019)

People with experience of using opioids for PNCP were recruited using posters displayed in GP practices, community pain services and community pharmacies across the West and East Midlands and Wessex in the UK, and online by regular posts and paid advertisements using social media. For ease of reading, the term ‘patient(s)’ will be used to represent all the patient and public participants with experience of using opioids for PNCP who took part in this study.

Advertisements directed potential participants to the PROMPPT discussion forum with links to a participant information sheet, electronic consent form and registration form. Upon registration, participants were assigned an anonymous username. To ensure acceptability and accessibility of the discussion forum, software and interface were user tested by members of Keele’s Patient and Public Involvement and Engagement (PPIE) group.

One of 10 topics was released weekly over 11 weeks (supplementary material 3). Facilitation prompts were posted approximately daily to aide discussions.

Findings were discussed with the intervention development team and the PROMPPT stakeholder group and key intervention components were agreed for the prototype intervention ready for in-practice testing.

#### Experienced acceptability

Pharmacists from three general practices in the West Midlands were recruited to IPT (November 2019 -February 2020), with adult patients (>18 years) prescribed opioids for PCNP, recruited from electronic practice records. Pharmacists attended an afternoon of face-to-face prototype training at Keele University, including practising PROMPPT reviews with simulated patients.

IPT comprised three iterative cycles of delivery, data collection, reflection, and revision of the PROMPPT review. Patients were asked to think-aloud during the review by saying out loud any thoughts or feelings as they came up. Reviews were audio-recorded and observed by two qualitative researchers (NC, CW, TH, SW). Immediately following each review, the patient and pharmacist were interviewed separately by one researcher, using a TFA-informed topic guide (supplementary material 4) and probes related to observing the review. Interviews were audio-recorded. Researchers categorised observations into six categories; visual cues, verbal cues, think-aloud, resources, potential changes required and other things to note.

Interview and observation data from each cycle of IPT were combined with observations highlighting aspects that worked well as well as identifying areas needing revision. Mini-optimisations were then made that were evaluated during the next cycle.

### Data analysis

We used the TFA to understand the prospective and experienced acceptability of the PROMPPT review, including discussions about tapering opioids, among patients and pharmacists. To ensure rigour, a phased approach to analysis was adopted. Interview transcripts, including in-practice interviews, were professionally transcribed verbatim, anonymised and checked for accuracy against interview recordings. Online discussion forum posts were downloaded into Microsoft Excel® and forum user IDs replaced with deidentifying codes to further protect participant anonymity.

Transcripts were read and re-read for data familiarisation. A coding manual based on the TFA domains was created and discussed and an initial thematic framework developed using a priori theoretical constructs. Three transcripts were coded independently by a multi-disciplinary team including qualitative researchers, pain specialists and pharmacist (NC, CJ, CW, SH, SW) using NVivo v12 software to aid data management. The team met to discuss data and understanding of theoretical constructs before a second coding phase, followed by another coding discussion meeting. Two coders (NC and CW) then coded all remaining data (including discussion forum data) into the framework and ‘key aspects’ were generated within each TFA domain.

### Ethics

Ethical approval was granted by the East of England – Cambridge East Research Ethics Committee (ref:19/EE/0151). Informed written consent was obtained from all participants.

## Results

### Participant characteristics

#### Prospective acceptability

Twenty-eight semi-structured interviews were conducted, with patients (*n* = 15) (see Table 2 for demographics) (mean length 37 mins) and pharmacists (*n* = 13) (mean length 49 mins). Of the 13 pharmacists, 9 were female and 4 male.

**Table 2.** Patient interview characteristics.

Patients prescribed opioids for PNCP (*n* = 15)					
Gender	Age	Strength of opioid prescribed^a^			Total
	*mean years (range)*	*Weak*	*Intermediate*	*Strong*	
Male	68.75 (55–83)	1	1	2	4
Female	70.73 (54–87)	2	4	5	11

^a^Opioid strength grouping based on a published categorisation for prescribed analgesics in primary care.^15^

The online discussion forum recruited 69 participants, posting 160 comments. As online discussion participants joined and participated anonymously, full participant demographics are unavailable.

#### Experienced acceptability

Thirteen patients and 3 pharmacists from 3 UK General Practices participated in IPT. (See Table 3 for demographics). Two Pharmacists (GP practices one and two, both female) were involved in IPT cycles one and two, with the third pharmacist (GP practice three, male) involved in IPT cycle 3. Fifteen PROMPPT reviews (13 initial and 2 follow-up) were observed, with the patient and pharmacist interviewed following each consultation (patient interviews *n* = 15 (mean length 23 mins), pharmacist interviews *n* = 15 (27 mins)).

**Table 3.** In-practice testing (IPT) participant characteristics.

Patients (*n* = 13)								
IPT cycle	Gender	GP practice			Strength of opioid prescribed^a^			Total
		1	2	3	Weak	Intermediate	Strong	
1	Male	1	2	—	1	1	1	**3**
	Female	1	—	—	1	—	—	**1**
2	Male	1	1	—	—	1	1	**2**
	Female	1	2	—	2	—	1	**3**
3	Male	—	—	1	—	1	—	**1**
	Female	—	—	3	3	—	—	**3**
**Total**		**4**	**5**	**4**	**7**	**3**	**3**	**13**

^a^Opioid strength grouping based on a published categorisation for prescribed analgesics in primary care.^15^.

### Acceptability of PROMPPT: Patient perspectives

Patients talked about aspects of acceptability across all TFA constructs, apart from the domain of opportunity costs after experiencing the prototype PROMPPT review. The key findings are summarised in Table 4. Further illustrative data are provided in supplementary tables (supplementary material 5-8). Below we provide exemplars from each TFA domain:

**Table 4.** Summary of patients’ perceptions of acceptability of the PROMPPT review (*denotes aspect identified across all opioid strengths).

TFA constructs	Prospective acceptability of the proposed PROMPPT review	Experienced acceptability of the prototype PROMPPT review
Global acceptability	• Proposed reviews generally acceptable *	• Initial prototype PROMPPT review was helpful and enjoyable
		• Expect PROMPPT to be acceptable to most but not all patients
Affective attitude	• Positive attitude towards pharmacists*	• Pharmacists are approachable, qualified and knowledgeable*
	• Uncertainty about pharmacists and their role	• Knowledge of the pharmacist and their role and abilities helped the review
	• Gratitude for being invited for a review	• Grateful for participating in a PROMPPT review
	• Some patients fear having their opioids stopped	• Other patients will be scared and expect their opioids to be stopped
	• Patients value follow-up reviews	—
	• Uncertainty of the usefulness of a pre-review questionnaire to help prepare	—
	• Hope for the review varies amongst patients*	—
	—	• Prototype PROMPPT review well received and helpful*
Burden	• Engaging with a pharmacist for a PROMPPT review would be no effort*	• Engaging with the pharmacist was no effort
	• Location of the PROMPPT review affects how burdensome a review would be*	• PROMPPT reviews based at the GP practice reduced the burden
	• Tapering and withdrawal from opioids is difficult for patients	• Tapering can be difficult for patients who are not ready to change
	• Lack of trust in healthcare professionals and ‘blaming’ them for their situation of being on opioids will make engaging with a review difficult	—
	• Emotional aspects of pain are difficult to discuss	—
	• Level of burden is dependent on good or bad days with their pain	—
	—	• Pre-review questionnaire to help prepare was difficult to use*
Ethicality	• PROMPPT reviews need to be undertaken for the right reasons - to help patients manage their pain better*	• Important for pain reviews to review opioids*
	—	• Important to allow patients to choose when to be reviewed
Intervention coherence	• Understood the purpose of the proposed PROMPPT reviews*	• Understanding of what PROMPPT was aiming to achieve*
		• Invitation made clear what the purpose of the prototype review was
	• Understanding the proposed PROMPPT review components	• Patients understand the prototype PROMPPT review components*
	• Recognised need for a holistic review*	• PROMPPT reviews allow patients to feel valued and supported*
		• Appreciated the dedicated time and the collaborative approach
		• Understand that time is needed to allow PROMPPT reviews to work*
	• Belief that PROMPPT reviews will have additional benefits	• Cost saving for the NHS/GP practice is an additional benefit
	• Misunderstanding that the purpose of PROMPPT reviews is to find alternatives or won’t be suitable for them*	• Misunderstanding that the purpose of PROMPPT reviews is to find alternatives*
	—	• Don't feel prototype PROMPPT review was of value to them
Opportunity costs	• Patients place value on their opioids	—
Perceived effectiveness	• Optimistic that the proposed PROMPPT review will be successful in achieving its aims*	• Prototype PROMPPT review successful at reducing opioids
		• Prototype PROMPPT review has exceeded expectations*
		• Patient education empowered the patients
	• Effectiveness will be dependent on patient factors (i.e. dose, strength and readiness to change)	• Effectiveness will depend on the patient and their openness to discuss and engage in the review*
	• Some reservations that the intervention won’t be effective	• Scepticism of PROMPPT review and what it can achieve*
		• Helpful to reduce but not stop opioids
	—	• The review benefits the research and the pharmacist more than the patient
Self-efficacy	• Confident to discuss their pain with a pharmacist*	• Patients confident to participate in the PROMPPT review
	• Mixed confidence in their ability to reduce opioids	—

#### Affective attitude

When discussing their feelings towards the proposed PROMPPT review, patients were generally positive towards a pharmacist delivering the review. Some patients were uncertain of the role of a practice pharmacist, as they had not heard of them or consulted one previously. After experiencing a PROMPPT review, most patients felt that pharmacists were knowledgeable and qualified to deliver a review. Patients with prior knowledge of practice pharmacists and their role seemed more inclined to engage with the pharmacist during the review.

*‘I hadn’t heard of a clinical pharmacist until now…if I got to see a regular pharmacist and had plenty of time to discuss my pain and ways to deal with it, I think I’d be happy to see one.’* (Forum participant_67)

#### Burden

Some patients discussed their lack of trust in healthcare professionals resulting from poor pain management experiences, often blaming them for being dependent on opioids that provide no relief.

*‘I’m pretty damn miffed that I’m in an avoidable position here. This is literally prescribed harm.’* (Forum participant_19)

Although this lack of trust and discontent could have affected patients’ willingness to engage with a pharmacist during the review, they found pharmacists were approachable and voiced little effort required to engage with them during IPT.

#### Ethicality

Overall, it was important to patients that PROMPPT reviews are undertaken for the right reasons, to help patients manage their pain better and not for cost savings.

*‘It would also help people to have the purpose and aims of the appointment laid out and what to expect from the discussion - is it just to try to get me off opioids or a genuine desire to get my pain under control to help me - or to help the practice to cut costs, or to make their statistics look good’* (Forum participant_61)

Once patients had experienced the prototype review during IPT, they felt that other patients like themselves should expect to have their opioids reviewed and that, although it would be important to have set reviews, it would also be important to allow patients to choose when they want or need reviews.

#### Intervention coherence

There were mixed perceptions of the purpose of the proposed PROMPPT review with some patients believing it would be to review prescribed opioids, reduce them where appropriate and support them to live better with pain, whilst others expected to be given an alternative pain medicine to replace opioids. After experiencing the prototype review, some patients still felt that it was not suitable for them as they were only taking a weak opioid or low dose that did not warrant any change.

*‘If I was on a lot, I’d want to reduce it but I don’t take, I’m very careful what I do take.’* (IPT patient_5: female/weak opioid)

Once they had experienced PROMPPT, the majority of patients reported that the review left them feeling valued and supported and appreciated the dedicated time and collaborative approach to their pain management.

#### Opportunity costs

Some patients expressed concern about being invited and attending a review for fear of having their opioids stopped. They spoke of valuing opioids to help them get by day-to-day and feared stopping would impact on their priorities. This concern was not reported by any IPT patients once they had attended a PROMPPT review.

#### Perceived effectiveness

When discussing the potential of the proposed PROMPPT reviews, patients were generally optimistic that it would be successful, specifically in tapering down opioids, improving their quality of life and ability to manage their pain. However, some felt doubtful that a pharmacist would be able to help when a GP had not helped in the past. Some patients said the prototype review exceeded expectations, but some remained doubtful in how successful it would be in reducing their opioids completely, with a slight reduction seeming more realistic.

*‘I mean I would love to, to get rid of them all. Er, I can't see that happening er, in the future, being off them all but it might get reduced which would be good.’* (IPT patient_29: female/weak opioid)

#### Self-efficacy

Although most patients said they felt confident that they would be able to discuss their pain with a pharmacist, confidence in their ability to reduce opioids was mixed. Patients with tapering experience felt confident about the prospect of engaging in the opioid reduction process and generally reported feeling very confident participating in the prototype review during IPT, allowing them to engage and be open with the practice pharmacist during the review. However, those with no experience expressed uncertainty and nervousness about trying.

Perspectives of prospective and experienced acceptability seemed largely similar regardless of opioid strength used. However, when thinking about the proposed PROMPPT review there were differences in the domains of burden and self-efficacy. Those on strong opioids felt the review would be more burdensome if they were having a bad day and were less confident/more concerned about their ability to reduce. Following IPT those on weak opioids perceived that patients taking higher doses would be scared that their opioids would be stopped (affective attitude). They felt that tapering could be difficult for patients who were resistant to changing opioids (burden) and some felt it may be more realistic to reduce rather than stop opioids (perceived effectiveness).

### Acceptability of PROMPPT: Pharmacists’ perspectives

In prospective interviews pharmacists talked about aspects of acceptability across all TFA domains except in relation to ‘opportunity costs’. Key findings are summarised in Table 5. Further data are provided in supplementary tables. Below we provide exemplars from each TFA domain:

**Table 5.** Summary of pharmacists’ perceptions of Acceptability of PROMPPT.

TFA constructs	Prospective acceptability of proposed PROMPPT review	Experienced acceptability of prototype PROMPPT review
Global acceptability	• Proposed PROMPPT reviews will be acceptable to pharmacists	• Prototype PROMPPT review is acceptable
Affective attitude	• Pharmacists are qualified and perfectly suited to delivering PROMPPT	• Pharmacists should be involved in delivering PROMPPT
	• Structured approach to reviewing opioids is needed	—
	—	• Pharmacists liked and enjoyed the PROMPPT reviews
	—	• Pharmacist’s feelings towards the reviews dependent on the type of patient
Burden	• PROMPPT reviews will be challenging consultations	• Experienced challenging consultations
		• Level of burden dependent on type of patient
	• Navigating the patients’ relationships and preferences for other healthcare professionals	• Navigating other care that patients are receiving can be challenging
	• Additional time requirement to deliver PROMPPT reviews	• Did not require more time to prepare for PROMPPT reviews or to write-up clinical notes compared to other consultations
	• Emotional burden for pharmacists	• Emotional burden on pharmacists
	• Additional training requirement	—
	—	• Little effort is required to deliver PROMPPT
	—	• Patients knowing aims and being prepared made the review easier for the pharmacist
	—	• Accessing and referring to other services is often challenging
Ethicality	• Purpose should be to improve patient safety and quality of life	• PROMPPT’s motives are ethical
	• Opportunity to save money should not be a primary motive	
	• Need for consistency in reviewing opioids	• PROMPPT is important to allow consistency in reviewing persistent pain and opioids
	—	• Important for patient to feel supported
Intervention coherence	• PROMPPT aims to improve patient safety and quality of life	• Pharmacists understood the aims of PROMPPT
	• PROMPPT is about working in partnership with the patient and empowering them to manage their pain	• PROMPPT is about working in partnership with patients
		• PROMPPT is important to support patients and ensure they feel valued and their pain story validated
	• PROMPPT is a holistic and personalised review for the patient	• Importance of a holistic review for persistent pain
	• Understand the proposed review components	• Understanding inclusion of prototype PROMPPT review components
		• Acknowledgement that not all review components are relevant for all patients
	• PROMPPT will have additional outcomes, other than reducing opioids, for patients, pharmacists and the wider GP practice	• Acknowledgement for the additional cost-saving benefit of PROMPPT
		• PROMPPT provides job satisfaction, confidence in their abilities and builds their reputation within the GP practice
	—	• Follow-up reviews are important for both supporting the patients and providing feedback to pharmacists
	—	• Understanding that PROMPPT is not a one-off consultation but a longer term process with continued support for patients
Opportunity costs	—	• PROMPPT needs to fit with the patient’s priorities to enable engagement
Perceived effectiveness	• Optimistic that PROMPPT review will successfully taper down opioids	• PROMPPT was effective in achieving its aims
		• Optimistic that PROMPPT will be successful in the long-term
	• Optimistic that PROMPPT will improve care, safety and quality of life for patients	• Patients will see value in the review and will also feel supported and valued as a result
	• Success of tapering down opioids will be dependent on patient readiness to change	• Patient resistance will affect tapering success
	—	• The structure and review components allowed the consultations to flow, making it effective
	—	• Pharmacists felt they could or should have done more to make a difference
Self-efficacy	• Pharmacists need confidence to deliver PROMPPT and the individual review components	• Prior experience allows pharmacists to feel confident to deliver PROMPPT
	• Confident they will be able to make an impact no matter how big or small on the patient	—
	—	• Resistant patients can knock pharmacists’ confidence
	—	• Consultations skills are more important than having the clinical skills to deliver PROMPPT
	—	• Pharmacists not as confident in discussing self-care and non-pharmacological alternatives
	—	• Generally confident to make referrals but more knowledge on available services would help

#### Affective attitude

When considering the proposed new pain review, pharmacists expressed a need for a structured approach to reviewing opioids making them more acceptable to deliver.

*‘I think it would be very acceptable because if we’ve got like a structured approach then when we are reviewing these patients, we feel like we’ve done justice to them as well and we’ve done the appropriate review and gone through everything we need to for the safety of prescribing and management and safety for the patients as well.’* (Interview pharmacist_21)

After taking part in IPT, pharmacists expressed how they liked and enjoyed delivering the prototype PROMPPT reviews and confirmed pharmacists’ prospective thoughts that they should be involved in delivering PROMPPT as they are appropriately qualified and experienced.

#### Burden

Reflecting on their previous experiences of consulting with patients prescribed opioids for PCNP, pharmacists had an expectation that they would have to deal with resistance from some patients to make a change. There was a perception that these patients are often complex, with multiple health and social concerns which make consultations more challenging. Some IPT pharmacists confirmed this expectation when describing the challenging consultations experienced during IPT, with patients often having a different agenda and not wanting to discuss making changes.

*‘I think her expectation at that time initially was maybe thinking about more medication or different types of medication, rather than perhaps my agenda which was more of a like trying to reduce the medication. So it was a bit of sort of internal conflict right at the start.’* (IPT pharmacist_3)

Despite these predicted challenges, pharmacists felt that the prototype PROMPPT reviews required no extra effort compared to regular consultations and found they became easier to deliver with practise and experience.

#### Ethicality

Pharmacists agreed that dedicated review for opioid-treated patients with persistent pain was important, with most saying these patients should be reviewed regularly and consistently to improve patient safety and quality of life. After delivering prototype PROMPPT reviews, all pharmacists said it provided patients the opportunity to be fully informed about their health and access to support.

### Intervention coherence

Pharmacists felt the proposed PROMPPT review needed to be a holistic review, aligned with individual patient’s needs, allowing time to discuss pain stories and strategies for living well with pain. They spoke about working in partnership with patients to empower them to manage their pain and their opioids. Reflecting on the prototype reviews after IPT, pharmacists recognised that PROMPPT is not just a one-off consultation but a longer term process and highlighted the importance of follow-up reviews to provide continued support for patients and feedback to pharmacists.

*‘the useful bit was getting her back and seeing how it was working, rather than the unknown. You send them off and you don’t have a clue. You hope your plan comes to fruition, but it could’ve just gone completely pear-shaped* (IPT pharmacist_2)

#### Opportunity costs

One of the IPT pharmacists acknowledged the need for the PROMPPT review to fit with the patients’ priorities, to ensure best possible engagement from patients and the best chance of making changes.

#### Perceived effectiveness

Pharmacists were optimistic that the proposed review would improve patient care. Most expressed confidence about opioid tapering but highlighted that success would depend on the patient’s readiness to change.

*‘I think it will be very effective and I think it would definitely help, just purely because of the time that we have and that dedication that we can have’* (Interview pharmacist_21)

After delivering the prototype reviews, pharmacists felt that PROMPPT was effective in tapering opioids and improving patients’ ability to manage their pain and hoped it would improve their quality of life in the long-term.

#### Self-efficacy

Overall, pharmacists felt they would be confident to deliver the proposed PROMPPT reviews including discussing medicines, having honest conversations and communicating with patients. Creating management plans with patients was a skill some said they felt less confident with and highlighted this to be a training need. Prior experience in doing medication reviews, prescribing and discussing medicines helped pharmacists to feel confident to deliver the prototype review but consulting with patients they perceived as ‘difficult’ or ‘resistant’, negatively affected the pharmacist’s confidence.

*‘Started off quite confident and then as soon as I hit the brick wall I felt a little bit like, ‘Oh no, where do I go now?’ So my confidence sort of just took a bit of a knock halfway through.’* (IPT pharmacist_2)

## Discussion

This work is timely, given NHS England’s medicines optimisation aim to reduce inappropriate prescribing of potentially dependence-forming medicines. Structured medication reviews (SMRs) are a key component of this medicines optimisation strategy^16^ and it is expected that clinical pharmacists working in primary care will lead on and undertake SMRs.^17^ The prototype PROMPPT review is consistent with recently published NHS England guidance on optimising care for adults prescribed medicines associated with dependence^18^ and includes key recommended components of SMRs, namely: shared decision-making and a personalised approach to exploring the balance of safety and effectiveness of current treatment.^16^

We used a novel approach by applying the TFA early in the intervention development pathway, to explore acceptability of the proposed PROMPPT review (prospective acceptability) and the acceptability of a prototype PROMPPT review (experienced acceptability), within MRC phase 1 evaluation.^11^

Generally, the PROMPPT intervention was deemed acceptable by patients and pharmacists at both the prospective ‘in-principle’ phase and experienced prototype testing phase. Patients were grateful to be reviewed and for someone to listen and help them. Pharmacists felt that they were suited to deliver PROMPPT as they have the knowledge of pain medicines and the skills to consult with patients prescribed opioids for PNCP. Patients and pharmacists acknowledged that PROMPPT might not be acceptable to all patients, for example those who are fearful of having their opioids stopped or not being ready to make any changes to their pain management.

Key findings from the interviews (patients and pharmacists) and online discussion forum showed areas of the proposed PROMPPT review that were acceptable and also recommendations for changes or additional intervention content that could improve its acceptability prior to prototype testing. Findings from IPT informed intervention refinement including pharmacist training, prior to formal feasibility testing. Table 6 presents the key findings and associated TFA construct from both prospective (interviews and ODF) and experienced (IPT) phases of the study, along with associated recommendations for intervention content.

**Table 6.** Key findings and associated recommendations for PROMPPT from prospective and experienced acceptability.

	Prospective acceptability of proposed PROMPPT review		Experienced acceptability of prototype PROMPPT review	
TFA construct	Key finding	Recommendation for IPT	Key finding after IPT	Recommendation for feasibility study
**Global acceptability**	PROMPPT reviews generally acceptable to patients	Acceptable – continue with focus on pharmacist-led review	Initial PROMPPT review was helpful and enjoyable	Acceptable – continue with focus on pharmacist-led review
**Affective attitude**	Positive attitude towards pharmacists delivering PROMPPT	Acceptable – continue with focus on pharmacist-led review	Pharmacists are approachable, qualified and knowledgeable and should be involved in delivering PROMPPT	Acceptable – continue with focus on pharmacist-led review
	Patients uncertain about practice pharmacists delivering a review	IPT letter inviting patients to attend a review needs to introduce the role of the pharmacist	Patients uncertain about what pharmacists can do within the practice	More work is needed to raise awareness about pharmacists’ role and skills (independent prescribers). Refine invite letter and patient information sheet
	Patients are fearful of having their opioids stopped	IPT invitation letter needs to explain the aim of the review and provide reassurance that opioids won’t be stopped without discussions and agreement from the patient	Fear of having opioids stopped may stop others engaging with the review	More work to do to explain the purpose of the review. Refine invite letter and patient information sheet
**Burden**	Location of the review affects how burdensome a review would be to patients	PROMPPT reviews to be based at GP practice	PROMPPT reviews based at the GP practice reduced the burden	Acceptable – continue with reviews based at GP practice
	Lack of trust in healthcare professionals	Building rapport with patients needs to be highlighted as a key aspect of the review. Pharmacist training to include guidance on how to build rapport with the patient	—	—
	PROMPPT reviews will be challenging consultations	IPT pharmacist training needs to include examples of challenging consultations and how to approach these with opportunities to practise skills during role plays	Consultations were challenging with patients often asking divergent health questions	PROMPPT training to include examples of challenging consultations and how and when pharmacists should keep reviews focussed on pain
	—	—	Pharmacists unsure what to document on the ‘Pain review plan’	PROMPPT training to include examples of completed pain review plans
**Ethicality**	PROMPPT needs to be undertaken for the right reasons to help patients manage their pain better	Invitations, preparation tools and discussions with pharmacists need to make clear the motive for PROMPPT reviews	PROMPPT’s motives are ethical	Acceptable – continue with motives underpinning PROMPPT
**Intervention coherence**	Understood the purpose of PROMPPT – working in partnership to empower patients to manage their pain	Acceptable – no recommendation	Understood what PROMPPT was trying to achieve and the invitation made clear the purpose of the review	Acceptable – continue with invitation outlining purpose of review
	Additional outcome for the GP practice – potential for improvement across whole practice in management of persistent pain	Whole practice approach is required to ensure joined up care. Other clinical practice staff to be made aware of patients invited and attending PROMPPT reviews	Pharmacists worked in isolation during IPT, without other practice staff involvement. A whole practice approach was deemed important. How can PROMPPT help all practice staff understand and support the reviews?	A summary of the pharmacist training manual to be created and made available to practice staff including GPs to provide an overview of the PROMPPT study Guidance for administrative teams with frequently asked questions to be created
	—	—	Pharmacists needing to know when to ask for help and support from a GP	Guidance on when and how to approach GP for support to be included in the pharmacist training
	—	—	Follow-ups not routinely planned but importance of follow-ups for both patients and pharmacists was highlighted through interviews. How do we ensure that follow-ups are conducted?	Importance of follow-ups for patient support and pharmacist reassurance to be highlighted in the pharmacist training. Follow-ups are appropriate for all patients ‘Pain review plan’ to include space for details of follow-ups, to act as a reminder for the pharmacist. Also include space to write contact details for patients to feel supported following the review
**Opportunity costs**	Patients place value on their opioids (*patients)*	Pharmacists need to understand the reasons why a patient may not want to make a change to their opioids	—	—
	—	—	Pharmacists acknowledged that PROMPPT needs to fit with the patient’s priorities to enable engagement	Acceptable – continue to encourage pharmacists during training to allow the review to fit with the patient’s priorities
**Perceived effect** **iveness**	Optimistic that PROMPPT will be successful in achieving its aims and will successfully taper down opioids	Acceptable – continue to deliver reviews as planned	PROMPPT review exceeded expectations, was effective in achieving its aims and pharmacists optimistic that it will be successful in the long-term	Acceptable – continue to deliver reviews as planned
**Self-efficacy**	Patients confident to discuss their pain with a pharmacist	Acceptable – continue to deliver reviews as planned	Patients confident to participate in the review	Acceptable – continue to deliver reviews as planned
	Pharmacists need confidence to deliver PROMPPT and the individual review components – lack of confidence in making a management plan with the patient	Pharmacist training to include guidance on working together with a patient to create a management plan	—	—
	—	—	Pharmacists would benefit from having a better knowledge of available services to refer to	Encourage pharmacists to explore available referral services in their area in readiness for reviews

### Reflections on using the TFA

The TFA has been used at singular timepoints (prospective,^19,20^ concurrent^21^ and retrospective^22,23^) and across timepoints^24,25^ in intervention development and evaluation. We applied the TFA during the first phase of PROMPPT intervention development, including both prospective and experienced acceptability, allowing key concerns (about implementation or uptake) to be identified and addressed. Exploring prospective and experienced acceptability, highlighted how different constructs apply at different times. For example, patients expressed their concerns that PROMPPT reviews would need to be undertaken for the right reasons to help patients manage their pain and not be for NHS cost-saving when discussing ethicality. Following IPT, patients understood that the purpose of PROMPPT was to support them to live well with pain and that cost saving for the NHS and the GP practice was an additional outcome, demonstrating a shift from ethicality to intervention coherence.

Using the TFA to identify and address key concerns according to the different constructs, supports the assertion from Sekhon and colleagues that acceptability is not a fixed construct with binary (acceptable/not acceptable) outcomes. This is shared by Deja et al.,^25^ who acknowledged the merit in adopting the TFA to identify and address key issues that threatened the acceptability of their trial.^25^ The TFA was developed as a tool to help understand what makes health interventions acceptable and what needs to be addressed to improve its acceptability^12,13^ rather than defining criteria to assess whether something is acceptable or not according to set cut-offs. We used the construct of ‘Global acceptability’ as an overall marker of whether the intervention was acceptable or not and used other constructs to identify areas that required no change and recommendations for improvement. As no ‘red flags’ were identified in the key findings for each construct, we were reassured that the intervention was generally acceptable but could be improved by actioning the recommendations. We also observed a significant overlap in the constructs ‘Global acceptability’ and ‘Affective attitude’ when participants were discussing their feelings towards the PROMPPT intervention. This supported our decision to use ‘Global Acceptability’ as a marker to judge overall acceptability rather than to use it to identify aspects of acceptability as a separate construct. To support future research using the TFA, some clarification is required with regards to assessing acceptability and whether when an intervention or aspects of, can be classified as ‘acceptable’ according to the constructs.

### Strengths and limitations

We have adopted a multi-component approach using complementary qualitative research methods to support the early development phases of PROMPPT. Recruiting participants through a range of routes allowed us to capture perspectives from a wider range of people with experience of taking opioids for PNCP, including both those currently using opioids and those who had experience of tapering and stopping their opioids. Interviewing patients and pharmacists means that we have investigated potential problems for those either receiving or delivering the intervention. The study was further strengthened with the inclusion of prototype testing during IPT, allowing for potential barriers to implementation and issues of acceptability to be addressed to increase probability of intervention success.

During IPT, patients were asked to ‘think-aloud’ during the PROMPPT review by saying any thoughts or feelings that came up concurrently. However, none of them did this, and so we asked retrospectively about the review in the interviews afterwards. Concurrent think-aloud methods have been used previously in questionnaire design^26–28^ and intervention development studies.^29^ However, in the context of a primary care consultation, ‘think-alouds’ did not work so well as it disrupts the flow of the review and the dialogue between the patient and the pharmacist.

Issues highlighted during each stage of IPT were addressed where possible, but a short timeframe between each cycle limited how much could be addressed. Any recommendations not implemented during cycles (e.g. summary of pharmacist training for other practice staff including GPs and guidance for administrative teams) were addressed following the final cycle of IPT and helped to inform the intervention tested in a single-armed feasibility study to assess the acceptability and credibility of the pain management review.

## Conclusion

This paper highlights how assessing acceptability at multiple time points during early intervention development allows for refinement and development to optimise implementation in relation to acceptability.^12^ Using ‘Global acceptability’ as a marker, we were reassured of the overall acceptability of the PROMPPT intervention. Recommendations identified according to the TFA constructs allowed an acceptable intervention to be developed that was ready to be tested in a formal feasibility study. The TFA was used during feasibility testing and is now currently being used for evaluation of the intervention in a main cluster-randomised controlled trial as recommended.^12,13^

## Supplemental Material

Supplemental Material - Acceptability of a proposed practice pharmacist-led review for opioid-treated patients with persistent pain: A qualitative study to inform intervention development Acceptability of a proposed practice pharmacist-led review for opioid-treated patients with persistent pain: A qualitative study to inform intervention development by Nicola Cornwall, Charlotte Woodcock, Julie Ashworth, Sarah A Harrisson, Lisa Dikomitis, Simon White, Toby Helliwell, Eleanor Hodgson, Roger Knaggs, Tamar Pincus, Miriam Santer, Christian D Mallen, Clare Jinks: on behalf of the PROMPPT team in British Journal of Pain.

Supplemental Material - Acceptability of a proposed practice pharmacist-led review for opioid-treated patients with persistent pain: A qualitative study to inform intervention development Acceptability of a proposed practice pharmacist-led review for opioid-treated patients with persistent pain: A qualitative study to inform intervention development by Nicola Cornwall, Charlotte Woodcock, Julie Ashworth, Sarah A Harrisson, Lisa Dikomitis, Simon White, Toby Helliwell, Eleanor Hodgson, Roger Knaggs, Tamar Pincus, Miriam Santer, Christian D Mallen, Clare Jinks: on behalf of the PROMPPT team in British Journal of Pain.

Supplemental Material - Acceptability of a proposed practice pharmacist-led review for opioid-treated patients with persistent pain: A qualitative study to inform intervention development Acceptability of a proposed practice pharmacist-led review for opioid-treated patients with persistent pain: A qualitative study to inform intervention development by Nicola Cornwall, Charlotte Woodcock, Julie Ashworth, Sarah A Harrisson, Lisa Dikomitis, Simon White, Toby Helliwell, Eleanor Hodgson, Roger Knaggs, Tamar Pincus, Miriam Santer, Christian D Mallen, Clare Jinks: on behalf of the PROMPPT team in British Journal of Pain.

Supplemental Material - Acceptability of a proposed practice pharmacist-led review for opioid-treated patients with persistent pain: A qualitative study to inform intervention development Acceptability of a proposed practice pharmacist-led review for opioid-treated patients with persistent pain: A qualitative study to inform intervention development by Nicola Cornwall, Charlotte Woodcock, Julie Ashworth, Sarah A Harrisson, Lisa Dikomitis, Simon White, Toby Helliwell, Eleanor Hodgson, Roger Knaggs, Tamar Pincus, Miriam Santer, Christian D Mallen, Clare Jinks: on behalf of the PROMPPT team in British Journal of Pain.

Supplemental Material - Acceptability of a proposed practice pharmacist-led review for opioid-treated patients with persistent pain: A qualitative study to inform intervention development Acceptability of a proposed practice pharmacist-led review for opioid-treated patients with persistent pain: A qualitative study to inform intervention development by Nicola Cornwall, Charlotte Woodcock, Julie Ashworth, Sarah A Harrisson, Lisa Dikomitis, Simon White, Toby Helliwell, Eleanor Hodgson, Roger Knaggs, Tamar Pincus, Miriam Santer, Christian D Mallen, Clare Jinks: on behalf of the PROMPPT team in British Journal of Pain.

Supplemental Material - Acceptability of a proposed practice pharmacist-led review for opioid-treated patients with persistent pain: A qualitative study to inform intervention development Acceptability of a proposed practice pharmacist-led review for opioid-treated patients with persistent pain: A qualitative study to inform intervention development by Nicola Cornwall, Charlotte Woodcock, Julie Ashworth, Sarah A Harrisson, Lisa Dikomitis, Simon White, Toby Helliwell, Eleanor Hodgson, Roger Knaggs, Tamar Pincus, Miriam Santer, Christian D Mallen, Clare Jinks: on behalf of the PROMPPT team in British Journal of Pain.

Supplemental Material - Acceptability of a proposed practice pharmacist-led review for opioid-treated patients with persistent pain: A qualitative study to inform intervention development Acceptability of a proposed practice pharmacist-led review for opioid-treated patients with persistent pain: A qualitative study to inform intervention development by Nicola Cornwall, Charlotte Woodcock, Julie Ashworth, Sarah A Harrisson, Lisa Dikomitis, Simon White, Toby Helliwell, Eleanor Hodgson, Roger Knaggs, Tamar Pincus, Miriam Santer, Christian D Mallen, Clare Jinks: on behalf of the PROMPPT team in British Journal of Pain.

Supplemental Material - Acceptability of a proposed practice pharmacist-led review for opioid-treated patients with persistent pain: A qualitative study to inform intervention development Acceptability of a proposed practice pharmacist-led review for opioid-treated patients with persistent pain: A qualitative study to inform intervention development by Nicola Cornwall, Charlotte Woodcock, Julie Ashworth, Sarah A Harrisson, Lisa Dikomitis, Simon White, Toby Helliwell, Eleanor Hodgson, Roger Knaggs, Tamar Pincus, Miriam Santer, Christian D Mallen, Clare Jinks: on behalf of the PROMPPT team in British Journal of Pain.

## Data Availability

Supplementary materials for this article are available online including topic guides and illustrative quotes.
