# Supplementary material for: Acceptability of a proposed practice pharmacist-led review for opioid-treated patients with persistent pain: A qualitative study to inform intervention development
Source: Br J Pain. 2023 Dec 19;18(3):274–91. doi: 10.1177/20494637231221688 (PMC11092934; doi:10.1177/20494637231221688)
Supplement: Supplemental Material - Acceptability of a proposed practice pharmacist-led review for opioid-treated patients with persistent pain: A qualitative study to inform intervention development [file sj-pdf-1-bjp-10.1177_20494637231221688.pdf]

Q-PROMPPT: Qualitative study to design the PROMPPT intervention

Patient interview topic guide

1. Can you start by telling me a bit about your long-term pain?
2. What are your experiences of using regular medication and in particular opioids– things like codeine, tramadol, patches, oxycodone –for your long-term pain?
3. Have you had a review with the GP to discuss the medicines you use regularly to manage your long-term pain?
4. If you make an appointment to see the GP about your long-term pain – do you get asked about the regular medication you are using, including questions about opioids?
5. Have you ever talked to a health care professional about the idea of reducing the amount of opioids you take?
6. Do you know what pharmacists do? / Do you know what clinical pharmacists do in GP surgeries?
7. How would you feel about discussing your pain medicines with a clinical pharmacist?
8. If you had the opportunity to discuss your pain medicines with a clinical pharmacist what would you want this review to include?
9. How would you feel about making a plan with a clinical pharmacist to try to reduce the amount of opioid medicines you take for your long-term pain?
10. If you made a plan to reduce opioids, how would you feel about having ongoing discussions about this?
11. How would you feel about discussing benefits/side effects, taking alternatives, self-management, referral to other services, making a plan to reduce the amount of medicine you are taking?
12. Is there anything missing that you think should be added to a review of the medicines you take regularly to manage your long-term pain?
13. How willing would you be to discuss those things with a clinical pharmacist rather than a GP?
14. How confident do you think you would feel to discuss your use of pain medicines, including opioids, with a clinical pharmacist?
15. If you were invited to attend a review with a clinical pharmacist at your GP surgery - how likely is it that you would go?
16. How appropriate is this type of review?
17. How much effort would it take for you to see the clinical pharmacist and discuss how you use your pain medicines?
18. How fair do you think it is that patients with pain for longer than 6 months get invited to attend a review about their regular use of pain medication?

19. How will going to see a clinical pharmacist to discuss your pain medicines impact on your other priorities?
20. What problems do you think might occur if you go to the GP surgery for a clinical pharmacist review of your pain medicines?
21. How do you think attending a pharmacist review might impact on you?
22. How acceptable do you think a review like this will be to other patients like you who take regular opioid medicines for long-term pain?
23. What do you think a review with clinical pharmacists could achieve? What do you think the purpose of having this type of reviews is for patients with long-term pain, like you?
24. Do you think there will be any other health benefits from seeing a clinical pharmacist about the medication you use regularly for your long-term pain?
