# Supplementary material for: Acceptability of a proposed practice pharmacist-led review for opioid-treated patients with persistent pain: A qualitative study to inform intervention development
Source: Br J Pain. 2023 Dec 19;18(3):274–91. doi: 10.1177/20494637231221688 (PMC11092934; doi:10.1177/20494637231221688)
Supplement: Supplemental Material - Acceptability of a proposed practice pharmacist-led review for opioid-treated patients with persistent pain: A qualitative study to inform intervention development [file sj-pdf-2-bjp-10.1177_20494637231221688.pdf]

Q-PROMPPT: Qualitative study to design the PROMPPT intervention

Clinical Pharmacist Interview Topic Guide

1. Can you start by telling me a bit about the work that you do in GP surgeries?
2. What are your experiences of discussing, with patients, medicines used for long-term non-cancer pain, including opioid medicines?
3. Can you tell me about any recent experiences of talking to patients about reducing opioid medicines?
4. If you think about doing a review with patients about their regular opioids medicines use for persistent pain what would you want this review to include?
5. How do you feel about:
   - Suggesting they take alternative medicines instead of opioids?
   - Discussing self-management for managing persistent non-cancer pain?
   - Referring patients with persistent non-cancer pain to other services?
   - making a plan to reduce the amount of medicine patients are taking.
6. Can you describe to me what you feel may be the purpose of conducting reviews for patients taking long-term opioids for persistent non-cancer pain?
7. What are the benefits of doing a review like this?
8. Are there any disadvantages of doing a review like this?
9. Is there anything missing that you think should be added to a review that aims to reduce inappropriate prescribing of opioids in patients with persistent pain?
10. In general, how much effort do you think will be needed to do a review with patients about their regular use of opioids for persistent pain?
11. How confident do you feel that you can do a review and work with patients to reduce the amount of opioids taken regularly for persistent non-cancer pain?
12. How willing would you be to work as part of a multi-disciplinary team in a general practice to review patient’s regular use of opioid type medicines?
13. How appropriate do you think it is for you to work in general practice to review patients taking opioids for long-term non-cancer pain, with the aim of reducing inappropriate opioid use?
14. To what extent do you feel this type of review has ethical implications for patient care?
15. How do you think doing a review for patients prescribed long term opioids for chronic pain will impact on you?
16. How do you think doing a review for patients prescribed long term opioids for chronic pain will impact on patients?
17. How acceptable do you think a review like this will be to patients who using regular opioids for persistent non-cancer pain?

18. How acceptable do you think a review like this will be to your colleagues?
19. What do you think patients will think about this review of their opioid medicines?
20. How effective do you think this new approach using clinical pharmacists will be in reducing inappropriate use of opioids in patients with persistent non-cancer pain?
21. Do you think there will be any other health benefits for patients if they see a clinical pharmacist to review their use of opioids and reduce the amount being used?
22. That comes to the end of my questions. Is there anything else you would like to add about the idea of a clinical pharmacist reviewing patients in general practice to discuss patients' opioid use for chronic pain and reducing inappropriate opioids?
