# Supplementary material for: Acceptability of a proposed practice pharmacist-led review for opioid-treated patients with persistent pain: A qualitative study to inform intervention development
Source: Br J Pain. 2023 Dec 19;18(3):274–91. doi: 10.1177/20494637231221688 (PMC11092934; doi:10.1177/20494637231221688)
Supplement: Supplemental Material - Acceptability of a proposed practice pharmacist-led review for opioid-treated patients with persistent pain: A qualitative study to inform intervention development [file sj-pdf-3-bjp-10.1177_20494637231221688.pdf]

Online discussion forum topics

1. Living with long-term pain

- What is living with long-term pain like for you?
- How does pain affect your life?

2. Experience of taking regular medication for long-term pain

- What's your experience of taking regular pain medicines – now or in the past?

3. Talking to healthcare professionals about pain

- What opportunities do you get to talk with a healthcare professional (for example your GP or practice nurse) about your pain?
- What sort of help would you like from your GP or other healthcare professionals that you don't get at the moment?

4. Pain medication reviews – current practice

- Can you share your experience of a consultation (review) when you discussed your pain medication?

5. Pain medication reviews – how should it be done?

- How do you feel about having routine appointments to talk about your regular pain medication?

6. Experiences and views of clinical pharmacists

- Do you have any experiences you can share of clinical pharmacists working in GP surgeries?
- How do you feel about clinical pharmacists in GP surgeries reviewing patients on long-term pain medication?

7. Seeing clinical pharmacists about pain

- How do you feel about clinical pharmacists in GP surgeries reviewing patients on long-term medication?
- What thoughts might go through your mind if you were invited to discuss your pain with a clinical pharmacist?

8. Challenges of reviewing and changing pain medicines

- What thoughts and/or feelings would you have about changing your medicines?
- What would help you begin to think about making a change?

9. Self-care for long-term pain

- What has helped you to live better with pain?
- How did you find out about ways to live better with pain?
- How easy was it for you to find ways to live better with pain?

10. Attending pain reviews – the finer details

- What information in this letter would help you decide to make an appointment?
- What might put you off from attending an appointment?
- How acceptable would it to ask you to complete a short tick-box form (2 sides of A4) before the appointment?
