# Supplementary material for: Acceptability of a proposed practice pharmacist-led review for opioid-treated patients with persistent pain: A qualitative study to inform intervention development
Source: Br J Pain. 2023 Dec 19;18(3):274–91. doi: 10.1177/20494637231221688 (PMC11092934; doi:10.1177/20494637231221688)
Supplement: Supplemental Material - Acceptability of a proposed practice pharmacist-led review for opioid-treated patients with persistent pain: A qualitative study to inform intervention development [file sj-pdf-4-bjp-10.1177_20494637231221688.pdf]

Q-PROMPPT: Qualitative study to design the PROMPPT intervention

In-practice testing with 'think-aloud' interviews

Topic guide for Patient Interviews

1. Could you tell me in your own words what you thought the purpose of the review was today?
2. How did you feel about discussing your regular medication for persistent pain with the clinical pharmacist?
3. Was there anything in particular that you liked about the review?
4. Was there anything that you disliked about the review?
5. How did you feel about:
   - Discussing the pros and cons of taking these types of medication?
   - The idea of making changes to the regular pain medicines you are taking?

     o The idea of reducing the amount of opioid medication you are using for your pain?
   - Learning more about self-management for your pain?

And if applicable:

- Being referred to other services for your pain?
- Having follow-up discussions about reducing the amount of regular medication you take for your pain?

6. How willing were you to discuss the above aspects with a clinical pharmacist?
7. Which part of the review did you think was particularly useful /relevant?
8. Which part of the review did you find problematic or feel should be avoided?
9. Was there anything missing that you think should be added to a clinical pharmacist review for patients taking regular opioid medication for log-term pain?
10. Is there anything else about the review that you would change?
11. How confident did you feel participating in a consultation with a clinical pharmacist about reviewing / reducing the regular medication you take for persistent pain?
12. How much effort did it take to have this review with the clinical pharmacist?
13. Do you think it was appropriate for you to discuss your regular pain medication and make plan to change this with the clinical pharmacist?
14. How do you think this clinical pharmacist review impacted on you?
15. How has this review impacted on your other priorities?
16. How fair do you think it is that patients with pain for longer than 6 months get invited to attend a review about their regular use of pain medication?

17. What do you think this type of review could achieve? How effective do you think this new approach using clinical pharmacists will be in reducing how much regular medication you take for your persistent pain?
18. Do you think there will be any other health benefits from seeing a clinical pharmacist about your opioid painkillers?
19. How acceptable do you think this new way of reviewing people who take regular opioids is?
20. Just by way of a summary – what in general are your views about reducing the amount of regular medication you take for your persistent pain?

Topic guide for Pharmacist Interviews

1. Could you tell me in your own words what you thought the purpose of the review was today?
2. How did you feel about discussing regular opioid medication with the patient?
3. Was there anything in particular that you liked about doing the review?
4. Was there anything that you disliked about doing the review?
5. How did you feel about:
    - Discussing the pros and cons of taking these types of medication?
    - The idea of making changes to their regular pain medication?
    - The idea of reducing the amount of regular opioids they are using for persistent pain?
    - Talking about self-management for pain?
    - Referring to other services for pain?
    - Having follow-up discussions about reducing the amount of regular opioids they are taking for persistent pain?
6. How willing were you to discuss the above aspects with the patient?
7. Which part of the review did you think was particularly useful /relevant?
8. Which part of the review did you find problematic or feel should be avoided?
9. Was there anything missing that you think should be added to a review for patients taking regular opioids for persistent pain?
10. Is there anything about the review that you would change?
11. How confident did you feel conducting a consultation with a patient with persistent pain to review their regular medication and reduce this where appropriate?
12. How much effort did it take to conduct the review?
13. Do you think it was appropriate for you to discuss use of regular pain medication and make a management plan with the patient in the general practice setting?
14. How do you think the review impacted on you?
15. How fair do you think it is that patients with persistent pain who have taken regular opioids for longer than 6 months get invited to attend a review about their pain medication? Should patients be able to choose when they get a review?

17. What do you think this type of review could achieve - How effective do you think this new approach using clinical pharmacists will be in reducing inappropriate use of opioids by patients with persistent non cancer pain?
18. Do you think there will be any other health benefits that patients get from seeing a clinical pharmacist about use of pain medicines?
19. How acceptable do you think this way of reviewing people taking regular opioids is?
20. Just by way of a summary – what in general are your views about reducing the amount of regular medication patients take for persistent pain?
