# Supplementary material for: Acceptability of a proposed practice pharmacist-led review for opioid-treated patients with persistent pain: A qualitative study to inform intervention development
Source: Br J Pain. 2023 Dec 19;18(3):274–91. doi: 10.1177/20494637231221688 (PMC11092934; doi:10.1177/20494637231221688)
Supplement: Supplemental Material - Acceptability of a proposed practice pharmacist-led review for opioid-treated patients with persistent pain: A qualitative study to inform intervention development [file sj-pdf-5-bjp-10.1177_20494637231221688.pdf]

Supplementary File S5 - Table 1: Patient - Prospective acceptability summary of key findings

Key Finding	Supporting quotes
**GLOBAL ACCEPTABILITY**	
**Proposed reviews generally acceptable**	‘It’s essential [yeah]. Yeah, absolutely. If you’re on strong medication erm and you can’t see that there’s gonna be any difference or it’s gonna stop, you need to keep going back all the time to check that there’s no other problem getting created by taking these medicines.’ *Interview patient_15: Female/Intermediate opioid*<br><br>‘I think its a very good idea to have reviews it allows for problem solving with medication and also ideas to change to another if needed sometimes people put up with bad side effects not knowing what to do about it’ *Forum Participant_40*<br><br>‘Very appropriate because I think the GP’s are under that much pressure [yeah] it would be nice for to be able to sit and talk to someone different [yeah] you know erm…who is just, it’s something new to try isn’t it?’ *Interview patient_4: Female/Strong opioid*
**AFFECTIVE ATTITUDE**	
**Positive attitude towards pharmacists**	‘If I can talk to them as I can talk to my doctor [mmm] and they can erm, explain why I should be on the medication that is all I’m asking for.’ *Interview patient_1: Male/Strong opioid*<br><br>‘Well I think I would be happy to talk to someone who has an in-depth knowledge [ok] of medicines and sometimes, I don’t want to put GP’s down, but sometimes I think they have an overall knowledge of a lot of things [yeah] and it’s nice to talk to someone who is a specialist [ok yeah] so I think I’d be quite happy if I thought they were a specialist and they properly knew about side effects and long term side effects.’ *Interview patient_20: Female/Weak opioid*<br><br>‘I would welcome speaking to a pharmacist about pain control/meds. Have greatest respect for them and after all they are the people who have studied them in depth and work with the issues every day’ *Forum Participant_61*
**Uncertainty about pharmacists and their role**	‘I'm not sure whether a clinical pharmacist would understand, from my perspective all the illnesses that I've got; the complications…I'm not sure whether they would be - whether I would feel quite as confident in the pharmacist as I do my doctor because I've got a very good doctor.’ *Interview patient_14: Female/Intermediate opioid*<br><br>‘The thing is, the GP sees everything about me, pain management practice, mind, etc, and meds as a whole, whereas I'd be very concerned that seeing a med specialist for pain would isolate one part and take it out of context.’ *Forum Participant_22*

	‘I honestly don’t know. Erm, whether a clinical pharmacist could achieve anything a doctor hasn’t achieved, I wouldn’t like to say’ *Interview patient_22: Female/Weak opioid*
**Gratitude for being invited for a review**	‘I think it’s a really good idea, I’d value the opportunity, if I needed to, to have someone I could talk to without thinking no I have to be ill to go and talk to them if you see what I mean, an acute illness.’ *Interview patient_20: Female/Weak opioid*<br><br>‘As long as you I’ve got someone that I can sit down and I’m not – I haven’t got a timed seven and a half minutes or whatever it is and we can discuss it, I would be quite happy.’ *Interview patient_1: Male/Strong opioid*<br><br>‘But in principle in think it’s a very good idea, why not specialise, there could be advantages if they are the specialists, if they’d got a little bit more time and they’re going to be able to give more attention to it than the GP who’s got to know everything about everything and has got an enormous amount of pressure on them.’ *Interview patient_9: Male/Intermediate opioid*
**Some patients fear having their opioids stopped**	‘Am I gonna get withdrawal symptoms?...Well, I don’t want to jump about the bed like my husband does you know when he had his withdrawals erm I’ve had withdrawals erm with the dihydrocodeine and it’s horrible and I think that would be my upper most worry you know is the withdrawals…Erm, I don’t know if I would be scared erm I’ve been that used to taking them for so long.’ *Interview patient_4: Female/Strong opioid*<br><br>I was really nervous about going the first time because I knew that he would want to try to reduce my pain medication and that's what happened’ *Forum Participant_21*<br><br>‘I think this needs to be done without alarming the patient and them being concerned their medication could all of a sudden be stopped’ *Forum Participant_17*
**Patients value follow-up reviews**	‘I think it would be very important you know because erm there are other treatments out there, herbal remedies you know it would be nice to be able to discuss other you know pain relief…I would like to see them at least once every three months…Yes, that would be very important if I was having any real – what’s the word I’m looking for? Any real problems then I could phone up and discuss it, you know.’ *Interview patient_4: Female/Strong opioid*<br><br>‘Oh, I think I would need to, yeah…that is actually what they do at our surgery. If you go onto a new one then you go back in two weeks or a month, depending what it is, just to check that everything’s as it should be.’ *Interview patient_15: Female/Intermediate opioid*<br><br>‘I was talking to a friend, who has also managed her chronic pain without meds, and we both said that since coming off all

	meds (2years for me) neither of us have been asked back for a review on how we are coping!!' *Forum Participant_02*
**Uncertainty of the usefulness of a pre-review questionnaire to help prepare**	'I would probably write down so many things so that I'm you know prepared. *Interview patient_4: Female/Strong opioid*<br><br>'Sometimes things come into your head and 'I must remember to do that' and I'm now in a position where I write everything down when I go to the doctors so I don't forget to ask the right questions anyway.' *Interview patient_15: Female/Intermediate opioid*<br><br>'Erm, I'm not sure that it'd help me very much, would it really? I don't know. You see, I would go there on the assumption that she thought that she could help me, So how do I prepare for that?' *Interview patient_19: Male/Weak opioid*
**Hope for the review varies amongst patients**	'Basically help you reduce a pain, maybe not get rid of it all, but to reduce it is better than nothing.' *Interview patient_5: Male/Strong opioid*<br><br>'I would be quite willing to see them initially, you know, because sometimes people, people have got things that, that - you know, that, that you've not tried.' *Interview patient_14: Female/ Intermediate opioid*<br><br>'I can't argue with the MRI scans that I've seen about my back. And I know, I know the situation it's in. So I don't feel any amount of talking is going to alter that' *Interview patient_2: Female/ Strong opioid*
**BURDEN**	
**Engaging with a pharmacist for a PROMPPT review would be no effort**	'Wouldn't be any effort…I'd, I'd gladly go.' *Interview patient_11: Female/Strong opioid*<br><br>'It would be the same effort as going to see my GP, it wouldn't be any different' *Interview patient_14: Female/Intermediate opioid*<br><br>'No it wouldn't be an effort, it would be fine' *Interview patient_20:Female/Weak opioid*
**Location of the PROMPPT review affects how burdensome a review would be**	'It all depends where it is.' *Interview patient_3: Female/Strong opioid*<br><br>'It's not… not a massive effort really no, because it's literally you know only up the road, if it was ten minutes away..if it weren't in the village and the doctors was five miles away it would be massive, it would be a big problem' *Interview patient_12: Female/Intermediate opioid*<br><br>'However, if it is at the local surgery, for me it is just a short drive. I have a car without which I would go nowhere as the bus

	stop is up a hill. Mobility and breathing problems - and I live in a rural area too.' *Forum Participant_61*
**Tapering and withdrawal from opioids is difficult for patients**	'I've tried it myself… I tried going onto one a day and erm I didn't get a very good night sleep after it. So I went back to two again after I tried it for a few days. I thought 'No, I'm going back to two'.' *Interview patient_19: Male/Weak opioid*<br><br>'For me I was ready for that replacement to be the app I used and I was in the right place to be supremely motivated to change significant things in my life, but it's challenging and scary and I'm in a privileged position that I have been able to concentrate on those lifestyle changes, not everyone has that. And it took me many years to get to that position. I still feel vulnerable that I have nothing I can "take" for my pain if it flares' *Forum Participant_05*<br><br>'So I'm incredibly motivated and I have no problems with wanting to get off the drug, it's managing the withdrawal effects and severity of them and how to cope with them…Now it's literally the fear of the withdrawal effects and experiencing them that are my biggest problems...I'm incredibly motivated...but I'm also incredibly scared of going through withdrawal again.' *Forum Participant_05*
**Lack of trust in healthcare professionals and 'blaming' them for their situation of being on opioids will make engaging with a review difficult**	'I can understand that prescribing the opioids was done with no ill intent. I can accept that the failure to assist in tapering was done with no ill intent, but that still counts as negligence to me. But for it to take so long to be heard, to be listened too, to access the help I need is extremely poor and it has left me with some major trust and respect issues.' *Forum Participant_05*<br><br>'It's hard to know who you need to turn to for support at those times, the ambulance service and doctors both leave you in a limbo where neither is willing to do a whole lot but neither wants to hand off responsibility entirely. So even if you're in a comfy time in life that limbo with no real support hangs around.' *Forum Participant_19*<br><br>'I have had great difficulties of talking to some medical professionals about pain. Some haven't even believed the amount of pain that I have been in "*Patient says her pain score is 8, but she is talking on her mobile phone*." Another example was, "*maybe she thinks differently to what we do*" (in response to pain. A further example was, "*maybe she needs to be seen by a psychiatrist or health psychologist and reviewed*.." There were clearly times in the past when nursing staff and others were making derogatory comments about my health and pain - or that I was simply disbelieved.' *Forum Participant_20*
**Emotional aspects of pain are difficult to discuss**	'It can make me very depressed.' *Interview patient_2: Female/Strong opioid*<br><br>'Sometimes it can be quite emotional and I struggle with that definitely' *Interview patient_12: Female/Intermediate opioid*
**Level of burden dependent on good or**	'It depends how I am on that particular day.' *P002 Strong Female*

**bad days with their pain**	‘Sometimes it depends if I’m having a good day or a bad day, I either have to cancel you see.’ *Interview patient_4: Female/Strong opioid*
**ETHICALITY**	
**PROMPPT reviews need to be undertaken for the right reasons – to help patients manage their pain better**	‘I think it’s a really good idea, I’d value the opportunity, if I needed to, to have someone I could talk to without thinking no I have to be ill [yeah] to go and talk to them if you see what I mean, an acute illness.’ *Interview patient_20: Female/Weak opioid*<br><br>‘We should be able to discuss openly more about pain medication’s, find out all the side effects and what they’re doing to our bodies you know. When you go for a review they just go through your tablets, ‘How are you doing on that?’ fine, that’s it you know done and dusted.’ *Interview patient_4: Female/Strong opioid*<br><br>‘I never had a review during the whole 12 years I was on opioids! The only time my meds were reviewed was when the GP couldn’t increase them further and sent me to the Pain Clinic and then my meds were discussed in depth with me and the discussion began about tapering and then stopping the opioids! I hope this is beginning to change!!’ *Forum Participant_03*
**INTERVENTION COHERENCE**	
**Understood the purpose of the proposed PROMPPT reviews**	‘I’d like to discuss exactly what the medicine is doing in the body you now the sort of physiologically exactly what’s happening and what effects it can have so explaining why it’s having side effects and what exactly they are and if they’re long term’ *Interview patient_20: Female/Weak opioid*<br><br>‘Well yeah because that’s what you know the appointment’s about really discussing the pain medication in all aspects…well in terms of if the pain medication that I’m taking is helping, what I feel that the side effects are and you know what’s the sort of length of time that they are thinking of having to take it.’ *Interview patient_12: Female/Intermediate opioid*<br><br>‘I think its a very good idea to have reviews it allows for problem solving with medication and also ideas to change to another if needed sometimes people put up with bad side effects not knowing what to do about it’ *Forum Participant_40*
**Understanding the proposed pain review components**	‘With untreatable, chronic pain, you have to learn that you're stuck with it - and the medical establishment can help by being honest.’ *Forum Participant_22*<br><br>‘Given that those with Chronic Pain are probably suffering pain way above normal in the first place - and have to put up with it day in, day out - just saying 'give me a rating on 1 to 10' is unhelpful: the question should be 'How much worse than your normal pain'. And understand that 'normal' pain can vary a lot depending on activity, meds, pain management, relaxation,

	time of day, emotions, state of mind, etc.’ *Forum Participant_22*<br><br>‘Reviews should include how to holistically manage pain with ideas of self-management, alternative therapies and suggested strategies to improve movement and fitness.’ *Forum Participant_37*
**Recognised need for a holistic review**	‘It's obvious to me, now, and after learning the hard way, that everyone's pain management regime has to be different, tailored to them, their capabilities and their pain - something the clinicians I saw never really emphasised.’ *Forum participant_22*<br><br>‘Often things can interact and have to look at the whole picture. You know or say the holistic sort of thing and that’s important’ *Interview patient_22: Female/Weak opioid*<br><br>‘I believe that any persistent or complex pain condition should be treated holistically and in a multidisciplinary manner...’ *Forum participant_5*
**Belief that PROMPPT will have additional benefits**	‘Ive found them very helpful i think its a good idea to talk to them instead of a doctor that allows the doctor to do more. I’ve found advice and sorting medication out very easy with a clinical pharmacist’ *Forum Participant_40*<br><br>‘I can see this having amazing benefits from the point of view it would free up GPs to deal with other less straight forward things.’ *Forum Participant_66*<br><br>‘Oh, I think it would achieve erm peace of mind and you know, things like that. And emotionally I think it would be good. Erm it’s erm, you know, to be able to get it off your chest and talk to somebody who knows and who understands erm and not feel a nuisance.’ *Interview patient_13: Female/Intermediate opioid*
**Misunderstanding that the purpose of PROMPPT reviews is to find alternatives or won’t be suitable for them**	‘I keep trying to leave my drugs off but I can’t but I don’t think that’s a big dose anyway’ *Interview patient_7: Female/Strong opioid*<br><br>‘You can have as many reviews as you like, but unless there’s an alternative you know, I don’t see what erm what point – there wouldn’t be much point in it really’ *Interview patient_19: Male/Weak opioid*<br><br>‘My feeling is that regular review of my pain meds although sounding like very good practice might achieve nothing. I cope (sort of) on the analgesia I have now…Changing medication can be disruptive. There may be side effects. Pain may not be as well controlled and then there is a period of having to get it all under control again.’ *Forum participant_61*
**OPPORTUNITY COSTS**	

**Patients place value on their opioids**	'As long as I could still get about and still run my home. Anything, you know. As long as I was pain-free. But what bit I do I can only do with the help of my medication.' *Interview patient_2: Female/Strong opioid*<br><br>'I'd have concerns you know, that I wouldn't be able to get about as much as I do erm because you know, I'm retired now and it's important to keep going isn't it and go places and whatnot. So that's the idea in retirement. Erm I wouldn't like to be in constant acute pain all the time' *Interview patient_13: Female/Intermediate opioid*<br><br>'We should not have opioid medication withdrawn from those that it works for - unless there is another alternative that works just as well.' *Forum participant_13*
**PERCEIVED EFFECTIVENESS**	
**Optimistic that the proposed PROMPPT review will be successful in achieving its aims**	'You know and if they can help you, it'd be wonderful…Well if I explained it to them and you work out a programme and it works, I think it'd be very beneficial.' *Interview patient_13: Female/Intermediate opioid*<br><br>'Get me off the drugs for a start with…I think it would, definitely because I just think people are so ignorant about drugs and they think they go to the doctors, get a pill and it's going to fix everything and it won't' *Interview patient_7: Female/Strong opioid*<br><br>'Oh, I think it would achieve erm peace of mind and you know, things like that. And emotionally I think it would be good. Erm it's erm, you know, to be able to get it off your chest and talk to somebody who knows and who understands erm and not feel a nuisance.' *Interview patient_13: Female/Intermediate opioid*
**Effectiveness will be dependent on patient factors (i.e. dose, strength, readiness to change)**	'It may be very, very helpful to people in the early stages of having this sort of problem. To guide them through it, but I think sort of old hands like me you know, we've heard it all before really.' *Interview patient_2: Female/Strong opioid*<br><br>'I don't know, it depends on the person really doesn't it?' *Interview patient_2: Female/Strong opioid*<br><br>'It depends on how they feel and how they look at their life really. Their lifestyle and what medication they take' *Interview partient_22: Female/Weak opioid*
**Some reservations that the intervention won't be effective**	'Probably not at all. No. Erm because of what it is, erm as I say, it doesn't go away. Some of the things that you can do don't get rid of it, but it does alleviate it slightly, supposedly. I've not come across anything yet, alternative that is going to make – has made any difference to me' *Interview patient_15: Female/ Intermediate opioid*<br><br>*INT: Do you think they'll be able to try and reduce the amount?*

	‘Erm, I don’t really know. I don’t think it would. Erm because obviously if you’re in pain, the first thing you want to do is not be in pain [laughs].’ *Interview patient_22: Female/ Weak opioid*<br><br>I honestly don’t know. Erm, whether a clinical pharmacist could achieve anything a doctor hasn’t achieved, I wouldn’t like to say [laughs]. *Interview patient_22: Female/ Weak opioid*
**SELF-EFFICACY**	
**Confident to discuss their pain with a pharmacist**	‘I’d be more than confident erm…it’s, you’ll do anything to get out of pain darling, anything.’ *Interview patient_4: Female/Strong opioid*<br><br>‘Yeah, fine. Yeah, I’d feel you know, fine. You know, no problem. Quite confident, yeah.’ *Interview patient_13: Female/Intermediate opioid*<br><br>‘No I’d be fine [yeah] I’d be confident.’ *Interview patient_20: Female/Weak opioid*
**Mixed confidence in their ability to reduce opioids**	‘See how things go er, with it and whether he’ll have to give me different painkillers to try and help me erm, through, yeah’ *Interview patient_1: Male/Strong opioid*<br><br>‘But I don’t know how I’d come off them ‘cause I, they’d have to start on me a low…Yeah. Have to go on like, I couldn’t go through that.’ *Interview participant_11: Female/Strong opioid*<br><br>‘I’m not worried about tapering the patch though as I’ve happily and safely done it before’ *Forum participant_5*
