# Supplementary material for: Acceptability of a proposed practice pharmacist-led review for opioid-treated patients with persistent pain: A qualitative study to inform intervention development
Source: Br J Pain. 2023 Dec 19;18(3):274–91. doi: 10.1177/20494637231221688 (PMC11092934; doi:10.1177/20494637231221688)
Supplement: Supplemental Material - Acceptability of a proposed practice pharmacist-led review for opioid-treated patients with persistent pain: A qualitative study to inform intervention development [file sj-pdf-6-bjp-10.1177_20494637231221688.pdf]

Supplementary File S6 - Table 2 Patient - Experienced acceptability summary of key findings

Key Finding	Supporting quotes
**GLOBAL ACCEPTABILITY**	
**Initial PROMPPT review was helpful and enjoyable**	‘And you know if it’s helping it’s something…It’s helping me today…Well I keep thinking it’s helping people, well it’s helped me an awful lot this morning.’ *IPT patient_3: Female/weak opioid*<br><br>‘I think that's been very useful in the fact that we've, we've come to that erm, end today…Oh yeah, I, I saw some help for myself in there’ *IPT patient_27: Male/Intermediate opioid*<br><br>‘I found it very helpful and it’s been a nice way of spending the morning actually, it hasn’t been boring like I thought’ *IPT patient_30: Female/weak opioid*
**Expect PROMPPT to be acceptable to most but not all patients**	‘For me, it's entirely acceptable, yeah. We don't want people on drugs for the rest of their lives if they can help it, do we?’ *IPT patient_27: Male/Intermediate opioid*<br><br>‘It depends on the patient. I, I find it excellent. I'm not sure everyone would erm, but I think it's a good idea to try.’ *IPT patient_27: Male/Intermediate opioid*<br><br>‘To me I think it’s great, but I don’t know about everybody, some people might not like it especially if they think you know being monitored yeah.’ *IPT patient_33: Female/weak opioid*
**AFFECTIVE ATTITUDE**	
**Pharmacists are approachable, qualified and knowledgeable**	‘I think she dug quite deep, yes. It’s nice to know somebodies bothered rather than just putting up with it and shoving in more pills…Just talking in depth to somebody who feels equivalent to a GP in this respect.’ *IPT patient_11: Female/Strong opioid*<br><br>‘I just felt quite relaxed with him really and easy to talk to yeah you know he understood, I think he understood how I felt and that was it yeah…I thought it was great yeah because he understands the medication and obviously, he’s got more time that the GP. I’ve never actually discussed it with the GP.’ *IPT patient_33: Female/weak opioid*<br><br>‘So she really said carry on pretty well the same, but we’ll try just a slight experiment with the paracetamol…it made sense. I could follow her thinking and reasoning…I’m happy about that because she’s obviously said it from experience of having done it

	before.' *IPT patient_12: Male/Intermediate opioid*
**Knowledge of the pharmacist and their role and abilities helped the review**	'Because I think when like I've come to something like this I think because [CP Name]'s sort of like been involved in it he's got more involved in it, I think if I might have seen him, I don't know about him though I've seen him before and he's very chatty and very helpful but if it had been another doctor or something I don't think…[*You would have got as much from it*] No, probably not, no.' *IPT patient_30: Female/weak opioid*<br><br>'She made it clear that she could change it [*prescription*] and I was happy about that. Though a little surprised…Yes, I was delighted that could be done straight away' *IPT patient_12: Male/Intermediate opioid*
**Grateful for participating in a PROMPPT review**	It's made me feel that there's somebody there now so I can ring up when I am bothered and say, 'Can I come and see you?'…it's ever such a comfort to know that erm she's there' *IPT patient_3: Female/weak opioid*<br><br>'I was quite happy, when I first got the letter' *IPT patient_33: Female/weak opioid*<br><br>'Well, I mean if you have problems then you know you can always ring up can't you but it's nice to know that their keeping their eye on you…Yeah, you're not been forgotten, yeah.' *IPT patient_33: Female/weak opioid*
**Other patients will be scared and expect their opioids to be stopped**	'It's acceptable to me, but you might have somebody else in who won't cause they're losing their crutch, 'you're going to take them off me, what am I going to do?'…but if you've got somebody who's become addicted to them, they're not going to bother, they won't contact you cause they'll lose it, if they think they're going to lose it, they won't contact you, they'll leave it as long as possible.' *IPT patient_13: Male/Weak opioid*<br><br>'Old people are not going to want to take part because they're going to think, 'They're going to take some of my medication off me.' And there's a lot of people, I would think, that would be frightened er, to come off any kind of pain medication in case they get wo-, - more pain. They're, they're not willing to try it and I can understand that as well. *IPT patient_29: Female/Weak opioid*<br><br>'You'll probably get a lot of people not turning up because they probably soon click onto it, am I going to have my pills taken, so they might come but it would be 'oh it's too bad today' they cancel it. If they think you're going to cut them down, they don't want that.' *IPT patient_27: Female/Weak opioid*
**Prototype PROMPPT review well received and helpful**	'I think it covered things very well in quite a logical order erm, and he was ready to explain everything, even though sometimes I didn't need the explanation, you know, but other people might. So I think he covered everything superbly.' *IPT patient_27: Female/Weak opioid*<br><br>'All of it, yeah. Yeah, I'm not just saying that, but I found it myself personally very good, I'm glad I come' *IPT patient_30:*

	Female/weak opioid<br><br>'Yes, it's a bit deeper because you're doing more, you're looking at all of this and you're looking at everything, there's more time, more time for questions, more time for answers, GP, you've got to be very quick….the time benefit's much better.' *IPT patient_4: Male/Strong opioid*
**BURDEN**	
**Engaging with the pharmacist was no effort**	'It's been good it's just been quite straight forward and easy.' *IPT patient_3: Female/weak opioid*<br><br>'It was an easy conversation I thought really. No effort at all. It was easy.' *IPT patient_12: Male/Intermediate opioid*<br><br>'Nothing was too difficult to discuss and if I didn't understand anything, he did explain it, yeah' *IPT patient_29: Female/weak opioid*
**PROMPPT reviews based at the GP practice reduced the burden**	'No effort getting here' *IPT patient_30: Female/weak opioid*<br><br>'Finding somewhere to park on the car park...No, no effort really.' *IPT patient_27: Female/Weak opioid*
**Tapering can be difficult for patients who are not ready to change**	'She's got her work cut out if she's going to try and get people off a lot of pills.' *IPT patient_5: Female/weak opioid*<br><br>'I mean if you're going down this road of putting people down on their pills, you've got your work cut out' *IPT patient_5: Female/weak opioid*<br><br>'I mean I wouldn't take them if I - I've tried not taking them. It doesn't work. That doesn't work' *IPT patient_14: Female/weak opioid*
**Pre-review questionnaire to help prepare was difficult to use**	'No, just some of the questions are a bit middle line aren't they so you don't know whether to tick it or not to tick it.' *IPT patient_4: Male/Strong opioid*<br><br>'I suppose the majority of people you deal with, around here especially, are old age pensioners. Erm, the bit about continuing or return to work, yeah erm…as I say that's quite erm, this thing about see family and friends, I can't see what's that got to do with opium' *IPT patient_13: Male/Weak opioid*

	‘Those are the questions that I think some people must tick far more than I did. I just ticked the one I think.’ *IPT patient_12: Male/Intermediate opioid*
**ETHICALITY**	
**Important for pain reviews to review opioids**	‘Probably a good way cause some people may overdo it. Some people might take extra on top of what they're doing which you know as well as I do can be dangerous.’ *IPT patient_4: Male/Strong opioid*<br><br>‘That medication should be reviewed more often’ *IPT patient_11: Female/Strong opioid*<br><br>‘You can’t – you can’t have too much concentration on things like that. So, a surgery that has this ability to do this, well and good.’ *IPT patient_12: Male/Intermediate opioid*
**Important to allow patients to choose when to be reviewed**	‘Although if it's not, not a set thing, say, on a particular date, it should be a set thing that we're looking at reviewing regularly and then leave to choosing the date to when you, around the time you want to see people.’ *IPT patient_27: Female/Weak opioid*<br><br>‘I think you should be able to have an appointment when you need it [yeah] and not just when they think you should come.’ *IPT patient_30: Female/weak opioid*
**INTERVENTION COHERENCE**	
**Understanding of what PROMPPT was aiming to achieve**	‘I believe that behind it all there is an attempt to improve patient care with regard to painkillers and things like that.’ *IPT patient_12: Male/Intermediate opioid*<br><br>‘It was looking at what the problem was; seeing what the medication could do about that and making sure we weren't actually just accepting the medication for what it was and doing nothing about changing the dosage’ *IPT patient_27: Female/Weak opioid*<br><br>‘Because when you go for a medication review, it's just a quick review, because the, the time isn't there. So with [*Pharmacist*] like that, he can explain things better…Yeah, I found it very informative and er, reassuring, if you like; that it's, it's hopeful that it can be reduced…cause I, I am concerned about the co-codamol’ *IPT patient_29: Female/weak opioid*
**Invitation made clear what the purpose of the prototype review**	‘The fact that she, I mean it’s open, I know what it’s for so’ *IPT patient_13: Male/Weak opioid*<br><br>‘I didn’t expect any other really because the letters I’ve received or the information I’ve received has been overstating almost that you know, if you don’t like what’s going on, just say so and then you can walk out if you want to’ *IPT patient_12:*

**was**	*Male/Intermediate opioid*<br><br>'I think that the invitation to come was so carefully worded that you didn't feel you were being imposed on.' *IPT patient_12: Male/Intermediate opioid*
**Patients understand the prototype PROMPPT review components**	'I think it should be assessed cos we just learn to live with it…you can do a check up on the need for the drugs cos you get in the habit of taking them and that's it.' *IPT patient_11: Female/Strong opioid*<br><br>'If there's ways that can help besides the stuff that we're taking which is what she mentioned, if there are things that can be done that can make things better…she put forward things that were interesting, some not for me cause I'm okay, erm, but for other people, perhaps that's a good idea, because some people don't do enough' *IPT patient_4: Male/Strong opioid*<br><br>'You've got to educate people and probably try and do other things, take their mind off the painkillers…Its got be more you see than just tablets' *IPT patient_5: Female/weak opioid*
**PROMPPT reviews allow patients to feel valued and supported**	'It's made me feel that there's somebody there now so I can ring up when I am bothered and say, 'Can I come and see you?' I know you can ring up the pharmacist, I've got one here now, that if I'm mithering about something I can ring her and feel that it's all in confidence.' *IPT patient_3: Female/weak opioid*<br><br>'It's all very helpful to know somebody's interested.' *IPT patient_11: Female/Strong opioid*<br><br>'It's better this time…I think it's because he decided to do it rather than the doctor said, 'You must do it,' sort of thing… No, it was just suggestions. So er, that's what I thought about, you know. I thought, well, if your doctor tells you, "Oh, you must do this," then it makes you a bit tricky...and then you're anxious, and then it defeats the object, doesn't it? But yeah, doing it this way, I'll definitely have a go.' *IPT patient_29: Female/weak opioid*
**Appreciated the dedicated time and the collaborative approach**	'Yes it's a bit deeper because you're doing more, you're looking at all of this and you're looking at everything, there's more time, more time for questions, more time for answers' *IPT patient_4: Male/Strong opioid*<br><br>'It had got a purpose to it in which I was involved rather than going along and taking my problems along and seeing what they could do about it.' *IPT patient_27: Female/Weak opioid*<br><br>'I just felt that he understood what was going on, and he was concerned about the problems I was having and he was looking for ways to help me which would probably help others in the future, you know' *IPT patient_27: Female/Weak opioid*
**Understand that time is needed to**	'Yes, give it time to work and see.' *IPT patient_11: Female/Strong opioid*

**allow PROMPPT to work**	‘It's, it's something to bounce back er, you know, see how I've been feeling and...Yeah, see if it's worked, yeah’ *IPT patient_29: Female/weak opioid*<br><br>‘I think it would – it would prove to be beneficial, if in a few months’ time I can say, ‘I’m glad you changed one of my pills because I’m not getting so much pain’.’ *IPT patient_12: Male/Intermediate opioid*
**Cost saving for the NHS/GP practice is an additional benefit**	It’s a pity they can’t do more often [yeah] cos we all take so many pills, you could cut the drugs bill in half.’ *IPT patient_11: Female/Strong opioid*<br><br>‘Some patients have been on some stuff for, opioids and tramadol and stuff and they’ve been on the same medication for 30, 40 years and its never been picked up, which I can understand that. Erm and I think that’s part of it. Erm, to me there's far too many drugs being used on the NHS as it is or unnecessary drugs. So if they can cut back on that yeah, all for it, so it.’ *IPT patient_13: Male/Weak opioid*
**Misunderstanding that the purpose of PROMPPT reviews is to find alternatives**	‘Well I know it was to do with the erm, the pain relief and stuff like that but erm, I don’t really understand quite what you’ve achieved out of this cause all I've done is sat and told you sort of my story and the Doctor said, oh yeah, this, that and bumf, given me this.’ *IPT patient_2: Male/Intermediate opioid*<br><br>‘To see if there's any better pain relief for me probably, I don’t know.’ *IPT patient_5: Female/weak opioid*<br><br>‘Oh, to gather information from erm I presume, a number of people who will have erm pain problems for some time and they are – they should be pretty good at knowing what triggers pain off or what tablets have been good for them and so on. And you can’t hear too much or too many people, cos every now and again you’ll hear something you haven’t heard before and it makes sense.’ *IPT patient_12: Male/Intermediate opioid*
**Don't feel prototype PROMPPT review was of value to them**	‘If I was on a lot, I’d want to reduce it but I don’t take, I'm very careful what I do take.’ *IPT patient_5: Female/weak opioid*<br><br>‘She mentioned coming off it, but that wouldn’t be a good idea cause I've tried that before, that’s not a good idea. But I wouldn’t go backwards for myself, I think quite a few might not either’ *IPT patient_4: Male/Strong opioid*
**OPPORTUNITY COSTS**	
**-**	-
**PERCEIVED EFFECTIVENESS**	

**Prototype PROMPPT review successful at reducing opioids**	'I'm not taking any morphine now.' *IPT patient_11: Female/Strong opioid*<br><br>'I felt great because I've been trying it on my own anyway in a very minor way.' *IPT patient_27: Female/Weak opioid*
**Prototype PROMPPT review has exceeded expectations**	'I didn't expect it to work so well.' *IPT patient_27: Female/Weak opioid*<br><br>'Yeah, I found it very informative and er, reassuring, if you like]; that it's, it's hopeful that it can be reduced…cause I, I am concerned about the co-codamol' *IPT patient_29: Female/weak opioid*<br><br>'I liked all of it really. I think it was erm well-directed, I thought it was significant erm and I hope it helps.' *IPT patient_16: Male/Strong opioid*
**Patient education empowered the patients**	'Well it can give them a bit more help if they don't know about it. I'll be perfectly honest, I think there's a lot of us that don't, until we're actually sent to a place, they discuss it, we don't even know, oh, is that what it is, that sort of thing. And then you realise' *IPT patient_4: Male/Strong opioid*<br><br>'I think you'll do well. I think its about education. That's what you've got to get through.' *IPT patient_5: Female/weak opioid*
**Effectiveness will depend on the patient and their openness to discuss and engage in the review**	'That depends on the patient erm, cause it's alright reducing it if they're better, but if they're reducing it and they're not, then you've put them through a ringer so but I still think you should review it. I don't have a problem with reviewing.' *IPT patient_4: Male/Strong opioid*<br><br>'It all depends on the individual, doesn't it?...You get somebody like me, I'm quite open to it, I mean I don't mind but you'll get some that are entrenched and they'll refuse and they'll think they're being picked on, you know, its victimisation so, as I say, that to me is not quantifiable because its erm, the individual person themselves.' *IPT patient_13: Male/Weak opioid*<br><br>'Yeah, well that's interesting. I think erm in my case it probably won't make any difference at all…Erm but I think in other people's cases it probably might make a considerable difference because of the you know, the depth of the interview process and all the rest of it. So nothing for me, but for somebody else I would think, yeah.' *IPT patient_16: Male/Strong opioid*
**Scepticism of PROMPPT review and what it can achieve**	'It is and it's what can I do for you today? Not a lot, because it's there isn't it? If you've, can you pull me leg, make me feel better in an instance, he can't can he?' *IPT patient_5: Female/weak opioid*<br><br>'I don't really understand quite what you've achieved out of this cause all I've done is sat and told you sort of my story and the

	Doctor said, oh yeah, this, that and bumf, given me this…Well I don't feel at the moment its done a lot for me, once I've tried these things out, if it does then fine but just answering a few questions doesn't do a lot does it?' *IPT patient_2: Female/weak opioid*<br><br>'To be honest with you, if the exercises haven't changed or there's nothing new on the horizon there, I very much doubt it. To be honest with you, you probably get more benefit from a nice, hot Turkish bath and a massage really' *IPT patient_16: Male/Strong opioid*
**Helpful to reduce but not stop opioids**	'I mean I would love to, to get rid of them all. Er, I can't see that happening er, in the future, being off them all but it might get reduced which would be good.' *IPT patient_29: Female/weak opioid*<br><br>'It's hopeful that it can be reduced.' *IPT patient_29: Female/weak opioid*<br><br>'I'm hoping it's going to be helpful. I mean you, you can't tell with just er, one consultation and then go away.' *IPT patient_29: Female/weak opioid*
**The review benefits the research and the pharmacist more than the patient**	'If it answers questions for your studies or her studies, then fine, it doesn't actually over do a lot for me.' ' *IPT patient_2: Female/weak opioid*<br><br>'...I would have thought erm, in the sort of long-term... er, and you talk to different people and how it affects them, then you can put your research to good use by thinking, 'Well, this pain medication isn't suitable for that type of pain' *IPT patient_29: Female/weak opioid*<br><br>'Well, it can confirm what the pharmacist has come to believe so far in her or his career. And can sometimes surprise them by what they hear, cos they haven't heard it before. So that gives them a chance to think about it and get it in perspective. So that you're gradually building up a knowledge about the subject you're describing or dealing with.' *IPT patient_12: Male/Intermediate opioid*
**SELF-EFFICACY**	
**Patients confident to participate in the PROMPPT review**	'Very, very confident.' *IPT patient_12: Male/Intermediate opioid*<br><br>'Entirely confident. I - er, I've always had a lot of confidence in medical practice' *IPT patient_27: Female/Weak opioid*<br><br>'Very confident, I'm quite a confident person, you've probably noticed yeah, I'm not frightened of saying what I think' *IPT patient_30: Female/weak opioid*
