# Supplementary material for: Acceptability of a proposed practice pharmacist-led review for opioid-treated patients with persistent pain: A qualitative study to inform intervention development
Source: Br J Pain. 2023 Dec 19;18(3):274–91. doi: 10.1177/20494637231221688 (PMC11092934; doi:10.1177/20494637231221688)
Supplement: Supplemental Material - Acceptability of a proposed practice pharmacist-led review for opioid-treated patients with persistent pain: A qualitative study to inform intervention development [file sj-pdf-7-bjp-10.1177_20494637231221688.pdf]

Supplementary File S7 - Table 3 Pharmacist - Prospective acceptability summary of key findings

Key Finding	Supporting quotes
**GLOBAL ACCEPTABILITY**	
**Proposed PROMPPT reviews will be acceptable to pharmacists**	‘I think it’s really what you need in primary care really is a dedicated pain management clinic [ok] erm so where you have, for example, like diabetes clinics, you have a pain management clinic.’ *Interview pharmacist_17*<br><br>‘I think it’s – well, it’s very up-to-date and, as I said before, it is a perfect clinic for er, pharmacists – primary care pharmacists whether they are prescribers or non-prescribers because it’s, it’s utilise your knowledge about medication for, for a clinic and I think that it’s one of the best things the pharmacists could do.’ *Interview pharmacist_20*<br><br>‘I think it would be very acceptable because if we’ve got like a structured approach then when we are reviewing this patients we feel like we’ve done justice to them as well and we’ve done the appropriate review and gone through everything we need to for the safety of prescribing and management and safety for the patients as well.’ *Interview pharmacist_21*
**AFFECTIVE ATTITUDE**	
**Pharmacists are qualified and perfectly suited to delivering PROMPPT**	‘So some of the pharmacists that work in the practices now have a better background of that kind of medicine management and an understanding of opioids and the issues around pain management so I can’t see there being any problems with it, I can only see there being positives around pharmacists taking the lead’ *Interview pharmacist_16*<br><br>‘I think pharmacists are the expert on the sort of, you know, the pharmacology and the drugs, so I think you know they kind of see the pharmacist as somebody who knows about medicines and therefore we’re well equipped to tell patients about the side effects and the long term effects of using medication so I think in that sense, as a profession, they’re quite well-respected’ *Interview pharmacist_17*<br><br>‘I think it’s – well, it’s very up-to-date and, as I said before, it is a perfect clinic for er, pharmacists – primary care pharmacists whether they are prescribers or non-prescribers because it’s, it’s utilise your knowledge about medication for, for a clinic and I think that it’s one of the best things the pharmacists could do.’ *Interview pharmacist_20*
**Structured approach to reviewing opioids is needed**	‘If we had like a structured, like a structured review I think it would fit in very nicely because at the minute we review a COPD patient and hypotension patient, we have a plan, we have care plans set up. And they flow very nicely because patients understand that these are the relevant questions or information that we need from them. And so they’re used to it because these care plans have been here always and they understand that these are important information for their wellbeing and the

	safety of the medication, obviously monitoring their condition. Where right now we don't have nothing so it's kind of you obviously you have a little tick list in your mind seeing 'what have I gone through the following things'. And make sure that you've gone through it all and you've got all the important bits and then asking the patient, you know, what they want from that medication.' *Interview pharmacist_21*<br><br>'I think it would be very acceptable because if we've got like a structured approach then when we are reviewing this patients we feel like we've done justice to them as well and we've done the appropriate review and gone through everything we need to for the safety of prescribing and management and safety for the patients as well.' *Interview pharmacist_21*
**BURDEN**	
**PROMPPT reviews will be challenging consultations**	'But if you get ones that have been on it for so long and like, no well you're not going to take anything away from me, I don't think they would accept this review. Er, even if you're saying, well actually you know let's explore different pathways, let's explore alternatives if possible. I think that would be very difficult and it would probably may not engage or find it acceptable.' *Interview pharmacist_21*<br><br>'And absolutely, er, tapering down, when we have a discussion can be difficult because, you know, there's always going to be the hesitancy and reluctancy. Er, and I think so I think whilst we're good as pharmacists in terms of perhaps knowing what to do, having those consultations can be difficult, especially when you get that really difficult or aggressive, how do you work around those. And that only comes with experience, you can't read a book or go into a lecture and prepare, you just need to get on and do I think. Er, and then you'll know, get a thicker skin I suppose.' *Interview pharmacist_23*<br><br>'Er, I would say a lot of my pharmacist colleagues are finding pain a little bit more challenging because it's not as prescriptive as the other long term conditions, so you're trying to get a drug to a certain dose and, you know, you check the blood pressure, you titrate the drug up, or you check the adrenal function and you titrate the drug up and there's a clear objective markers, you know, pain is much more subjective and, er, some of my colleagues are feeling this is much more of a psychological role that they're having, rather than a pharmacy role. So some people struggle with that and also the fact that some patients have complex problems so, er, some people are a bit worried about opening a can of worms and not being able to deal with it.' *Interview pharmacist_22*
**Navigating the patients' relationships and preferences for other healthcare**	'Erm, in my surgery most of the patients would be absolutely fine with it, but as I've said there are a few that might say I'd rather discuss that with the Doctor but the majority of them would be fine.' *Interview pharmacist_21*<br><br>'You will get a handful who'd want the Doctor to say that its fine but not many, given the extra time for people erm, the GPs just don't have the time and erm, if the clinical pharmacist can demonstrate they know what they're talking about and can offer the

**professionals**	time erm, and a sympathetic ear, then I think they should be as effective as anyone else' *Interview pharmacist_5*<br><br>'That's been very challenging but that's because one of the GP's has started these patients on Oxycodone who no longer works here so the patients build up a rapport with the GP and obviously have a trust with that GP and then they are coming to see a pharmacist and they think oh well the GP thinks it's fine, who are you pal, erm and it's the only thing that keeps the pain at rest, you know that kind of conversation' *Interview pharmacist_16*
**Additional time requirement to deliver PROMPPT reviews**	'Erm, so workload, it would probably reduce the amount of the patients I could see if I've got several of these one-hour long appointments, initially, then that's going to reduce the amount of other people I can see. Erm, but yeah, there's not going to be that many patients, they're only going to have the initial appointment once so after that it'll go back to a 20-minute appointment.' *Interview pharmacist_19*<br><br>'Yeah, I think to be done properly you'd need more consultation time erm, how it would impact on me, I cant see my practice you know, or what would happen to all the rest of my work so, you'd almost need another session really to do it erm, I don't think I could fit it in my allocated time because its already you know, its already allocated to all the diabetes stuff and the costs I need to collect and the support I need to do for the nurses and things like that, so erm, I can't see, yeah' *Interview pharmacist_5*<br><br>'I mean general practice is really stretched so it will take a long time and they take repetitive appointments, and they are difficult and – but if you put the effort in you get the rewards.' *Interview pharmacist_14*
**Emotional burden for pharmacists**	'I think it can be quite stressful, you could get patients who could be quite rude erm they could be, you know, they could be quite negative towards you, they might see that you are not making things better for them you are making things worse, so I think in that sense it could be emotionally quite draining.' *Interview pharmacist_17*<br><br>'And it is sometimes emotionally draining because a lot of these patients do have complicated lives and will also suffer from mood disorders, depressive type illnesses as well so there'll be a whole catalogue. But I guess that's about having, I tend to do a mixture of appointments so I find it difficult to do whole pain for the whole afternoon. So whereas a lot of patients, people might do that, they just have a pain clinic that afternoon, I think that's quite hard because you feel drained at the end of it. Whereas mine tend to get mixed around by other patients, er, so I will do a mixture of clinics within my clinic, er, so I'll cover pain or cover post MI patients or whatever. Er, so I don't tend to just do pain, er, for a session so yeah but I do think you, yeah some of those patients are emotionally draining for the clinician so you have your own self awareness of your own emotional state as well.' *Interview pharmacist_14*<br><br>'I think it can be quite demanding and yeah because of it's it can be quite draining I think emotionally, er, for the clinician. Which I found, er, mostly because, you know, there's other stuff going and you have to catch yourself.' *Interview pharmacist_23*

**Additional training requirement**	'Erm, yeah, would be quite a bit of effort, I think initially to get trained up so that you're confident in dealing with these consultations' *Interview pharmacist_19*<br><br>'Well prior to that I would require the tools, knowledge and all the stuff that I was going to provide the patient with' *Interview pharmacist_16*<br><br>'And, er, I don't think there's a lot out there, you know, specifically, I'm talking more holistically, or if there is it's expensive, £200, you have to take annual leave time, you know, er, which I'm not going to get funded. So it's finding the resources that's difficult.' *Interview pharmacist_23*
**ETHICALITY**	
**Purpose should be to improve patient safety and quality of life**	'Erm, so basically the prescribers, they are, one of the rules is to do no harm, so we've got to make sure that that is always at the forefront they're not putting them at risks by prescribing these medicines erm, and erm, so that's one ethical, ethical consideration' *Interview pharmacist_5*<br><br>'There's a lot of information, er, about how long term opioids, er, aren't always in the patient's best interest and they do cause more problems and side effects, er, than – and it is in NICE and the SIGN guidelines about that. Er, and also that, er, it is inappropriate and it's not ethical to keep ramping the dose up on an opioid if it isn't actually helping with their pain and it is actually causing more problems than at that point. Er, so I do think that there's, yeah it isn't just a case of when we do the med reviews just clicking on re-issue, it's got to have a very careful thought process. Because if they have a fall from that opioid or driving issues and they haven't been informed, it is a very, er, important area.' *Interview pharmacist_8*<br><br>'I think it's probably ethical to give people the opportunity to speak about their medicines if that, er, something that's useful to them. I think maybe they don't realise that it's worthwhile having that discussion, some people might not think it's relevant that at this moment in time that I think it is probably still ethical to have the conversations so that they're aware of any kind of risks that are associated with it.' *Interview pharmacist_22*
**Opportunity to save money should not be a primary motive**	'I think the main focus has got to be improvement of quality of life and safety…we've got to make sure that the priority is the patient's care not just cost.' *Interview pharmacist_19*<br><br>'I think erm, if the patient thought it was purely a cost saving review then that might be a bit dodgy' *Interview pharmacist_19*<br><br>'So I think well no, cost saving is a side effect of it, I think the main focus has got to be improvement of quality of life and safety.' *CP019*

**Need for consistency in reviewing opioids**	'I think it should be part of the norm, I don't think it should be unacceptable so I think in a good practice it should be being done anyway. Er, but to the level we want to do it to decrease it is going to be more intense potentially but, er, you know, all practices should be reviewing the medicines they're prescribing at least once a year so it's not – we're just doing it properly is what I would say.' *Interview pharmacist_14*<br><br>'I think it's really what you need in primary care really is a dedicated pain management clinic, erm so where you have, for example, like diabetes clinics, you have a pain management clinic'. *Interview pharmacist_17*<br><br>'I think it would be very acceptable because if we've got like a structured approach then when we are reviewing this patients we feel like we've done justice to them as well and we've done the appropriate review and gone through everything we need to for the safety of prescribing and management and safety for the patients as well.' *Interview pharmacist_21*
**INTERVENTION COHERENCE**	
**PROMPPT aims to improve patient safety and quality of life**	'Is to improve, er, their quality of life and also the safety. The opioids have been shown to have quite detrimental effects long term. And I think our main aim, and all healthcare professionals, is patient safety and that they're getting the best out of their medication, er, and that they can live the life they want to live really, er, pain free hopefully.' *Interview pharmacist_8*<br><br>'Hope to improve the patient's, ability to function or, improve their pain long term or help them to cope with their pain and also root out any underlying issues, er, or depression or coping strategies or stress that could be adjusted and exercise, diet, er, and link it in with physio perhaps and the, er, support services.' *Interview pharmacist_8*<br><br>'Safety – safety, making sure the patient understands the limits of these medications, potential side effects, when they need to refer to if they feel anything. Er, and if there's any change in their condition, overall safety, safety. Because a lot of the times patients, I just think they're not aware particularly when they're coming in with constant pain. Are they aware, are they forgetting because I've come across somewhere that they're forgetting things. Er, so safety has to be the biggest – safety has to be the biggest thing.' *Interview pharmacist_21*
**PROMPPT is about working in partnership with the patient and empowering them to manage their pain**	'Erm, yeah I think all of it really, the patient needs to feel that they've been included on all of the decisions and it needs to be something that they're going to be able to do, erm they don't want to feel like it's happened to them, they want to feel they've been the one to decide things, so yeah, the plan needs to be discussed with the patient and erm, what's realistic for them to be able to attend if they're housebound, they're not going to be able to get to physio and things like that so the plan has got to fit in with what the patient can do really.' *Interview pharmacist_19*<br>'I'd probably say the patients pace as well so I tend to give them a person centred approach and partnership with the patient, say where they are in their cycle of change and readiness to change. And kind of taking that approach like you're working with them

	and they feel as if you are working with them rather than you telling them what to do. So I think that kind of collaborative approach with the patient is key and then I think probably everything else follow on behind that.' *Interview pharmacist_22*<br><br>'Its called empowerment techniques and you basically get the patient to say what they want to do or why they want to do it and then you talk about, well what are your barriers or why aren't you doing it basically in a counselling sort of way, and they list the barriers why they're not doing these sort of things. Then you talk about each of those barriers and they then decide how they move forward pass the barriers and you make a treatment plan and sort of go on from there really.' *Interview pharmacist_23*
**PROMPPT is a holistic and personalised review for the patient**	'Yeah I mean I think clinical pharmacists, you know, they're not just about the opioids, that's part of the process so they would look at everything related to their medicine but also think about all those other holistic things hopefully that you can offer.' *Interview pharmacist_14*<br><br>'I think the benefit is that the pharmacist will tend to see the patients for like 20 minutes, half an hour so you're going to have a longer session with patients and you can have a more holistic approach and you would be just spending that time signposting them to different things.' *Interview pharmacist_22*<br><br>'But when I always review these patients, I deal with the patient's agenda first before I look at my own agenda' *Interview pharmacist_6*
**Understand the propsed review components**	'I think ideally all of them. I think the most important are that, er, that they know what to expect from the medication and what realistically, how much reduction in pain relief in the pain they can get. Er, side effects are very important and safety and monitoring as well ongoing, er, again ideally you'd want to cover everything really with them.' *Interview pharmacist_8*<br><br>'Yeah, I think sometimes I do medication reviews and you start talking about individual medicines, people will say, well thank you very much, no one's ever explained that to me before and I think there are definitely advantages of clinical pharmacists during the reviews, so if they have a better understanding of why they're prescribed things and erm what the overall aim of taking it then you know, the compliance is better and that would go with opioids as well if they think, oh I didn't realise that there was this risk or that risk actually erm, this is how intolerance works or erm, if its normal to experience a certain degree of pain or maybe I can do this, that and the other to maybe reduce my pain erm, rather than just take all these tablets, so yeah.' *Interview pharmacist_5*<br><br>'Erm, sort of pain is not in isolation erm, so erm, and the reasons you know, what might be affecting the pain erm, or the different depth, their perception of pain and what they like about taking their medicines or dislike about taking the medicines.' *Interview pharmacist_5*
**PROMPPT will have additional**	'I think by having a pharmacist helping the patient and the GPs, that will actually help because it might reduce some of the workload for GPs. *Interview pharmacist_18*

**outcomes, other than reducing opioids, for patients, pharmacists and the wider GP practice**	'Erm, cost for the NHS because if its not effective anyway and they also might be being prescribed drugs to combat side effects like laxatives erm, so I think there's a cost point of view' *Interview pharmacist_19*<br><br>'And one of my colleagues is talking about, er, one of the patients was an older man and his daughter came in and said, you know, thank you so much we've got our dad back because he managed to get the patient off their fentanyl patches or a lower dose so she had noticed quite a big difference' *Interview pharmacist_22*
**OPPORTUNITY COSTS**	
-	-
**PERCEIVED EFFECTIVENESS**	
**Optimistic that PROMPPT will successfully taper down opioids**	'I think it can have a massive impact, er, and I think that is – those patients that are already on it we can probably reduce half of those patients, to be realistic, by a significant amount, so I think with time and work that can be done' *Interview pharmacist_14*<br><br>'Erm I think, well I think from my own experience I think it's been effective because we have managed to reduce patients on opioids, so we know we have managed to get some patients off high dose opioids' *Interview pharmacist_18*<br><br>'I think it will be very effective and I think it would definitely help, just purely because of the time that we have and that dedication that we can have, again compared to the GPs in the GP world.' *Interview pharmacist_21*
**Optimistic that PROMPPT will improve care, safety and quality of life for patients**	'So I do think there's a lot of potential for improving their condition and, er, their daily sort of functioning so I do think it's got massive potentially really.' *Interview pharmacist_8*<br><br>'I think it can benefit the patients that they feel someone's actually reviewing them because quite often they just carry on and on and on for months or years and nothing's ever really changed or I think they might appreciate the fact that we're recognising that if things aren't effective then they might be better to change, so I think the patients would appreciate the fact that someone's actually taking an interest in them. Erm and that they might not realise that they feel ill because of the meds they might think it's because of the pain so it's a new approach really for them.' *Interview pharmacist_19*<br><br>'I think it would have, er, both short and term long term benefits, er, in that quite a lot of them as I've said they do feel that they're not really being heard or, you know, they've just sort of been put on opioids and left to get on with it, even though they're still in pain.' *Interview pharmacist_8*

**Success of tapering down opioids will be dependent on patient readiness to change**	'Getting patients off - I'm probably about 80% confident the pharmacists will try to support the patients. Er, I don't know, it depends where the patient is and probably none of the pharmacists have kind of stopped the medication without the patient being on board because not sure of the consequences of that.' *Interview pharmacist_22*
**SELF-EFFICACY**	
**Pharmacists need confidence to deliver PROMPPT and the individual review components**	'I think the main importance is the training and the competence and the confidence, I think it could be done by anyone in that role but we're in a good position because we've got a good broad knowledge of drugs so erm, yeah.' *Interview pharmacist_19*<br><br>I'm fine, I'm an independent prescriber and I'm quite happy to erm do that and I think it does depend on the confidence of the pharmacist, I mean a lot of pharmacists may do a review but then send the suggestion to the GP because they might not want to take the responsibility of doing it themselves so firstly I make my own judgement when prescribing so I don't ask the GP to prescribe if I've seen the patient.' *Interview pharmacist_18*<br><br>'I think it's officially part of our bread and butter seeing patients on any long term condition management, once these patients have been diagnosed I don't see any issue, as long as you've got the full competencies in place, to manage this, it's up to the individual to make sure they are competent or feel competent [yeah] but they should' *Interview pharmacist_16*
**Confident they will be able to make an impact no matter how big or small on the patient**	'I've come to live a little more comfortably with the fact that I can't necessarily solve their problems but I can potentially make a small part of their life a little bit better.' *Interview pharmacist_15*
