# Supplementary material for: Acceptability of a proposed practice pharmacist-led review for opioid-treated patients with persistent pain: A qualitative study to inform intervention development
Source: Br J Pain. 2023 Dec 19;18(3):274–91. doi: 10.1177/20494637231221688 (PMC11092934; doi:10.1177/20494637231221688)
Supplement: Supplemental Material - Acceptability of a proposed practice pharmacist-led review for opioid-treated patients with persistent pain: A qualitative study to inform intervention development [file sj-pdf-8-bjp-10.1177_20494637231221688.pdf]

Supplementary File S8 - Table 4 Pharmacist - Experienced acceptability summary of key findings

Key Finding	Illustrative supporting quotes
**GLOBAL ACCEPTABILITY**	
**Prototype PROMPPT review is acceptable**	'Very acceptable. Can't see any problems with it really' *IPT pharmacist_1*<br><br>'I think this erm – I think it's a very acceptable way. It's kind of a complete way of allowing the patient to give their opinion and their kind of perspective compared with if it's just reviewed on the desktop or it's just done as part of another review. So yeah, I think it's the only way it should really be done' *IPT pharmacist_3*<br><br>'I think they'd be quite open to it. I think they would be grateful to have some sort of a structure to you know, a pain review and all the resources that are available really. It just gives it a lot more credibility to be able to sort of explore the patient and not just focus on the medication side. So I think they'd be – yeah, they'd be really pleased to have that sort of training' *IPT pharmacist_1*
**AFFECTIVE ATTITUDE**	
**Pharmacists should be involved in delivering PROMPPT**	'A lot of the other clinicians in the practice don't necessarily have the time to spend talking about it. So I think it would be very – you know, it's very much worth pharmacists being involved in it, definitely' *IPT pharmacist_1*<br><br>'I think as a new resource in general practice I think we're kind of – it fits well for us to be involved in it. So I think yeah, we could – we've got the potential educational skills anyway or the background understanding to be able to take it forward' *IPT pharmacist_3*
**Pharmacists liked and enjoyed the PROMPPT reviews**	'This review was lovely, it was fine. I would say it was fine. Erm, so it was fine, I think it was a good thing for him' *IPT pharmacist_2*<br><br>'I think it's really useful…Yeah, it's really good, yeah' *IPT pharmacist_2*
**Pharmacist's feelings towards the reviews dependent on the type of patient**	'I suppose the pressure was off because I knew he was on such minimal analgesia anyway. So if it'd been the same scenario but he had been on a fentanyl patch, I probably would have been less relaxed' *IPT pharmacist_2*<br><br>'I'm just trying to compare to the last time really, but I think he was – it was good because he wanted to – he was recognising that he – he didn't think they were working and the whole testing thing' *IPT pharmacist_1*

	‘[*was there anything in particular that you liked about doing the review*?] No, not on this occasion. I found it really tough, really tough. I just thought I wasn’t gonna get anywhere. Flogging a dead horse’ *PIP002-IP004*
**BURDEN**	
**Experienced challenging consultations**	‘I think opening his mind to the non-drug aspects of managing pain. I think he’s – you know, in the back of his mind he did just want a miracle pill that’s just gonna take the pain away. And it was quite difficult actually, the aspect of thinking about when I mentioned the sort of mindfulness. He was like, ‘I could do that for five minutes and then I’ll get up and the pain was just the same’ and I was like, ‘Ah’ [laughter]. Not quite sure what to say to that. Erm [laughs] that was probably a bit of an awkward one actually’ *IPT pharmacist_1*<br><br>‘But actually the difficulty was actually there was nothing to find so actually some of the questions started to become quite closed because you were trying to find something to latch onto, some sort of motivational point to go, oh well he has constipation, oh well, he’s feeling sick with his tablets, there was nothing to, he’s not getting out and about, there was nothing to sort of latch onto because actually his condition is so well controlled, so that was probably the most trickiest part ‘*IPT pharmacist_2*<br><br>‘I think the more difficult bit was the point when she was talking about wanting to think about something that’s longer lasting. So again, the potential of having to escalate a dose or change it to something that perhaps I thought might be less appropriate, even though that’s what somebody else had already suggested to try from the pain clinic. So again, I think that was the most – that was more the awkward part of the consultation where you realise you’re gonna have to kind of make an argument for why that isn’t the best option really. And just making sure it’s kind of robust enough at that time to put them off and have something else to offer I think was the other hard side of that. *IPT pharmacist_3*
**Level of burden dependent on type of patient**	‘I suppose the pressure was off because I knew he was on such minimal analgesia anyway. So if it’d been the same scenario but he had been on a fentanyl patch, I probably would have been less relaxed, does that make sense knowing that he was on a lot of stronger medication’ *IPT pharmacist_2*<br><br>‘This took more effort [right], just cos you had to be a bit more articulate, formal’ *IPT pharmacist_2*
**Navigating other care that patients are receiving can be challenging**	‘So I felt it was a little bit more challenging to talk about pain because her situation, it was more – she was still more being treated for her pain. So she’s still under a specialist, she’s still obviously waiting for procedures, operations, erm currently having injections. So it kind of clouds your judgment about taking things away when they’re already kind of in that – in the midst of all that really erm and also potentially being managed by other clinicians even though her pain management is – comes undone’ *IPT pharmacist_3*<br>‘I think I feel more intimidated by the consultations where patients are still being seen by other services. Because it is, it’s that

	internal conflict between what you're gonna do and what they've already been told to do, told or been advised to do. And then the fact that they are gonna be reviewed again at some point by them and whether the information you give today sort of is in line with their agendas. So you've got like different agendas you're trying to meet. So it's not just yourself and the patient now you're trying to erm – trying to match, you're trying to also match other clinicians as well. So it's erm – so yeah, so that one felt quite difficult for that reason. So she's been to pain management and she said in [town district] previously and then she was transferred to [county area]. They tried to put her on an opioid already there, buprenorphine patches erm and then she's seen the osteo-surgeon recently regarding a knee replacement. So there's obviously a potential for a knee replacement, but not yet. So again, there's kind of like a pain management for a short – potentially shorter period of time. But I don't think it's just her knee is a problem anyway; she seems to have wider spread pain than that, so I think the pain management issue is more of a chronic concern. So anyway, it was just – it's just trying to manage all those with then the patient's perspective. *IPT pharmacist_3*<br><br>'I think the pain management clinic she'd been to was perhaps a concern. The fact that they'd already suggested opioids quite recently, well within the last six months. Erm so that was something I was going – I wanted to kind of sort of address, sort of see where she was up to on that. *IPT pharmacist_3*
**Did not require more time to prepare for PROMPPT reviews or to write-up clinical notes compared to other consultations**	'So yeah, it might take about – it'd probably take the best part of ten minutes really, cos it's quite a long consultation just to just think about what I've spoken about. Cos I haven't written much down, so I'll have to do it straight away as well. So erm – so yeah, it's normally – and it depends obviously how quick you are at doing – individuals, some write more than less, some might shorten things they say and things. So but as – if you look at all the clinical pharmacists, we tend to write quite lengthy consultation sort of notes. So erm yeah, it's quite – that part of it is a bit of a burden, but it's just part of every consultation unfortunately, but it does add a bit of time on afterwards' *IPT pharmacist_3*<br><br>'I had a quick look yesterday at his notes and jotted down his previous medical history and his drug history and I looked about his other conditions so i.e. his bloods, so it probably took me five minutes. So erm, I think the GPs would have probably looked about two minutes prior to the patient even coming in but I had a bit of an inkling that it might, I didn't know quite the format, its not like a standard consultation, so erm, I don't think it was unfairly biased in terms of prep compared to how we would do a normal surgery' *IPT pharmacist_2*<br><br>'I think as preparation-wise, very little effort. I think it was a little bit – it felt more effort during it. Just having to think on the ground. But like you know, as in preparation, not at all' *IPT pharmacist_3*
**Emotional burden on pharmacists**	'Outside of PROMPPT, you do. You take your work home with you, especially when things go badly. Probably the reality is you take the consultations that have gone badly home with you. And this is probably a consultation that I need to remember for those bad days' *IPT pharmacist_2*

	‘It was a bit heart-wrenching, his story. Cos it’s quite – you wanted to be empathetic, but you don’t want to sort of inflame the situation by going, ‘That’s really terrible isn’t it?’’ *IPT pharmacist_2*<br><br>‘So we talked about the empathy versus the professionalism. So that’s – that was the hardest bit’ *IPT pharmacist_2*
**Little effort is required to deliver PROMPPT**	‘I mean again it was all easy to do the consultation…It wasn’t a lot of effort’ *IPT pharmacist_3*<br><br>‘It didn’t feel like it was any harder than anything else that I do….Nothing – you know, not traumatic, not erm over-stretching me. Erm, I can’t really think of anything to say on that front’ *IPT pharmacist_1*<br><br>‘This review was lovely, it was fine. I would say it was fine’ *IPT pharmacist_2*
**Patients knowing aims and being prepared made the review easier for the pharmacist**	‘I liked the fact he knew exactly why he was here cause it made it so easy and he’d probably been able to think about why he’d been called in and what he was going to say’ *IPT pharmacist_2*<br><br>‘I think it’s always important to erm let the patient know what to expect. So I think it gives a bit of an idea from that and that sort of nice platform. And it sort of – well, it doesn’t big up the pharmacist role – but it sort of makes it more official’ *IPT pharmacist_1*<br><br>I feel the consultation went really well, I mean in terms of it just flowed didn’t it, erm, but I think it was just set up, the agenda was known, so actually there was no locking of horns so he knew why he was here, he knew why we were here, try to make it very clear that it was an open conversation rather than a purposeful, we’re going to do X, Y and Z and then it just allowed it to follow its pattern’ *IPT pharmacist_2*
**Accessing and referring to other services is often challenging**	So I could’ve brought her back for follow-up quite easily, but actually I thought it was more important that she went and had her physio. And to be honest, that might take six months’ *IPT pharmacist_2*<br><br>Yeah and you'll probably find clinicians are quite frustrated because actually we're not meant to have a postcode lottery, that was meant to have gone 10, 15 years ago but actually there is an inequality’ *IPT pharmacist_2*<br><br>‘Its difficult, it is difficult because actually you can’t really express that to your patient, erm and some patients are expert patients and will know that erm and they’ll challenge you about why are certain, why your area doesn’t offer such a service, their brother who’s 10 miles down the road, has the same condition and has been offered that service’ *IPT pharmacist_2*

ETHICALITY	
**PROMPPT's motives are ethical**	'Yeah absolutely so you've got somebody who's stable, not getting particularly any kind of serious side effects but again like you say it's about it's being managed over a number of years. So yeah you could easily just leave them alone and they can continue like that. But as you say that person then down the line is the person that you're trying to take off high doses of morphine because again they've been seen by another practitioner who's then added it on and yeah you just haven't been down a reduction path, or at least a holding path' *IPT pharmacist_3*<br><br>'And if it's just giving him the opportunity to think about it and talk about it rather than his diabetes or his blood pressure, which is things that go on in the background and generally people who don't – you know, they're quite used to kind of talking about having those managed, but pain medications, it's very – once you've started on them you are left just to manage it on your own. So I think that was useful. It felt useful today to talk about it with him and it's something he hadn't obviously done with anybody specifically for a while. Erm and again, hopefully reducing over the period of time his risk for further – further things that could you know, damage or impact his wellbeing' *IPT pharmacist_3*<br><br>'I think it's just equipping people with the knowledge and understanding to make those you know, choose that they're not taking – overtaking medication and they're fully aware of the risks and self-management' *IPT pharmacist_1*
**PROMPPT is important to allow consistency in reviewing persistent pain and opioids**	'Erm, I think its unfair that they don't attend. That's why I'm laughing. Cause actually it's really important erm, yeah, I don't think they do. You have the really extreme chronic patients that get referred into secondary care and the rest just seem to muggle through' *IPT pharmacist_2*<br><br>'Yeah and I think it's important. I think it's fair that they should be reviewed on what are potentially high-risk meds. Because we do it for other higher risk meds' *IPT pharmacist_3*<br><br>'But pain medications, it's very – once you've started on them you are left just to manage it on your own. So I think that was useful. It felt useful today to talk about it with him and it's something he hadn't obviously done with anybody specifically for a while. Erm and again, hopefully reducing over the period of time his risk for further – further things that could you know, damage or impact his wellbeing' *IPT pharmacist_3*
**Important for patients to feel supported**	'I think it was a good thing for him. Erm cause he obviously hadn't been seen for a while about predominantly his pain. He's been seen about his hearing, he's been seen about his IBD, he's been seen about his gout, but I know he hasn't been seen about his pain so for him it was really good' *IPT pharmacist_2*<br><br>'Any other health benefits? Hopefully sort of wellbeing in general and just the fact that they never got somebody that they can

	refer to if they have got any queries about their medication' *IPT pharmacist_1*<br><br>The other people are thinking, 'Do you know what, I keep hearing this on the news and I really wanna do something about it, but I'm not sure how' and probably just burying it under the carpet until somebody actually gives them an olive branch to help them out' *IPT pharmacist_1*
**INTERVENTION COHERENCE**	
**Pharmacists understood the aims of PROMPPT**	'It's trying to achieve erm, better health and wellbeing for the patient…it's about optimising their pain, their wellbeing and their health isn't it erm and it's a quick win, it's a set clinic to be able to do that' *IPT pharmacist_2*<br><br>'So the purpose of the review I would say is to try and minimise the use of opioid medications and to give the patient a bit more advice about self-care and what they can do for themselves to manage the pain better' *IPT pharmacist_1*<br><br>'Again it doesn't mean it's not a worthwhile conversation because perhaps it gives the patient a bit more mindfulness about going forward with a chronic pain condition, about reducing the risk of escalation and doses I suppose as well. So maybe there's more benefit just around that sort of idea, rather than reducing today' *IPT pharmacist_3*
**PROMPPT is about working in partnership with patients**	'So the training was very much about open questions, let the patient speak, erm, use some sort of motivational technique to try and get the patient to collaborate erm and change their current practice' *IPT pharmacist_2*<br><br>'Perhaps I don't need to be the person who suggests it. The fact that she suggested it is the better way realistically' *IPT pharmacist_3*<br><br>'I think it's really important to make sure that you're really focusing on what they're saying and listening to them. Cos it's really helpful to be able to draw on what they've said a bit later on in the consultation to sort of make a point about something and make it personal to them' *IPT pharmacist_1*
**PROMPPT is important to support patients and ensure they feel valued and their pain story validated**	'I suppose that's why we're here because patients need the support to perhaps, or the reassurance that that's a possibility for them to try isn't it. Hopefully if it's okay great, and if not go back to them. *IPT pharmacist_3*<br><br>'Any other health benefits? Hopefully sort of wellbeing in general and just the fact that they never got somebody that they can refer to if they have got any queries about their medication. Erm…Yeah, so you know, they're not gonna just cast out into the big, bad world and left to get on with it. They know they've got a point of contact and a practice to follow up with if they're having problems or need some further advice' *IPT pharmacist_1*

	‘Erm, patients having greater confidence in the practice pharmacist and sort of feeling listened to’ *IPT pharmacist_1*
**Importance of a holistic review for persistent pain**	‘But also looking at that holistic approach to pain and seeing if there’s anything else I could offer her that might just help her live better with the pain’ *IPT pharmacist_1*<br><br>‘It’s not just about one ailment, it’s not just about chronic pain, it’s everything else that’s going on in their lives’ *IPT pharmacist_2*<br><br>‘But it’s understanding the other aspects of what they’re managing. And to be honest, that’s kind of perhaps a different aspect of the role that we’re used to doing. It’s a little bit more away from the pharmacy side and more impacting patient management, which is more the clinical side isn’t it I suppose? So yeah, so for that reason it is different to what we might do normally’ *IPT pharmacist_3*
**Understanding inclusion of PROMPPT review components**	‘To discuss the lady’s longstanding history, going back 18 years of pain. And for me it’s more about pain and understanding it rather than about automatically – about medicines’ *IPT pharmacist_2*<br><br>‘She’s just a low-risk patient isn’t she? She’s not using it that much. But I suppose it’s always important to have those conversations, just so that she’s aware that in the future you know, she’s fully aware the risks of taking opioids. So that’s helpful’ *IPT pharmacist_1*<br><br>‘So I think - there should be some sort of action at the end, rather than just, ‘Thank you for coming’ and that’s everything done. I think there needs to be something that’s kind of followed through from it. And that’s a bit of a pressure again in terms of knowing you’re kind of building towards something, however small it might seem, just to give the patient that reassurance that they’ve come away from it with something more than they had when they came in. Because they can, that ultimately in itself is probably detrimental to their ongoing management isn’t it then? You need to kind of at least give them some – and there’s that hope that they’re being managed better in some way by – from the contact…Value. And whether that’s you know – it doesn’t have to be necessarily an improvement in their pain relief at that time. But at least it could be – like you say, well as we’re trying to do, the risk reduction over a period of time. So it might just be something that they just reflect on later and say, ‘Well yeah, somebody’s at least trying, they’re looking out for you’ basically’ *IPT pharmacist_3*
**Acknowledgement that not all review components are relevant for all patients**	‘So she’s not – I don’t feel like she’s really hammering the opioids. So I think it’s a difficult one to sort of – you know’ *IPT pharmacist_1*<br><br>‘It was difficult because she’s not taking very many. If she’d been taking full eight a day tramadol or Tramacet or whichever, erm I think I would’ve probably delved into that a bit more and asked her, ‘Do you feel like you’re getting any side effects?’. You

	know, somebody can feel a bit drowsy or constipated or you know. But again, just because of the amount she was taking, I just didn't really feel it was something I could explore too much because it just didn't seem too relevant' *IPT pharmacist_1*
**Acknowledgement for the additional cost-saving benefit of PROMPPT**	'A positive impact on patients isn't it really? Erm health professionals, the government will go on about 'Well it'll help other comorbidities, it's a financial cost-saving'' *IPT pharmacist_2*<br><br>'And there's also a cost-saving element as well isn't there, obviously. I mean outside of all that it's the use of more and more is gonna cost more and more, so' *IPT pharmacist_3*
**PROMPPT provides job satisfaction, confidence in their abilities and builds their reputation within the GP practice**	'It's positive, positive. Definitely positive experience, not just this particular patient, this consultation. I think the whole PROMPPT thing has been really positive…It just gives you more reassurance that you're doing an okay job' *IPT pharmacist_2*<br><br>'Hopefully I would see her for her medication review. Now she's met me, she might think, 'Oh yes, I'll speak to that pharmacist cos she was quite useful' and I think that would flow quite nicely' *IPT pharmacist_1*<br><br>'I would just say it's probably raised my profile again as a practice pharmacist' *IPT pharmacist_1*
**Follow-up reviews are important for both supporting the patients and providing feedback to pharmacists**	'Yeah so six weeks' time we sort of agreed on. So again I think that's the best, that's the best of that type of situation, I could hound her between now and then to say have you reduced it, have you reduced it, or are you ready to. But I think it's best to still put the ball back in their court because she's managed her pain for all these years, and again I don't, if she's willing to do it, and seemed quite willing at that point yeah I think it will ring a bell hopefully at that time. And then we'll still follow up and again if she hasn't then it might just be another opportunity just to sort of readdress and have a conversation about it and sort of go through her concerns of whether the time was right. So I think it's just good to have an opportunity to either follow up what's done, or at least have an opportunity to talk about ongoing' *IPT pharmacist_3*<br><br>'Erm the useful bit was getting her back and seeing how it was working, rather than the unknown. You sent them off and you don't have a clue. You hope your plan comes to fruition, but it could've just gone completely pear-shaped *IPT pharmacist_2*<br><br>'Whether it's a week or whether perhaps two weeks could be – I mean it doesn't really matter to be honest. But again, yeah I think it's just paramount that there's that contact. And again, it's difficult because again you've gotta then have appointments available to do that; even if it's not – and it doesn't necessarily I suppose have to be the same person. Ideally, there's continuity there, but I suppose in a practice where we have got like more than one pharmacist or similar, it's something that could be shared between practitioners. But ideally, yeah that same person, at least having that contact to say, 'How have you been?'. And

	that seemed absolutely natural to me to be honest. Cos that's something I would do usually if someone was on blood pressure medications, let alone something that's gonna have a bigger impact day to day on their life. Cos we kind of know it potentially could make their quality of life worse to begin with anyway. So I think that early contact for that reason is probably even more important, so' *IPT pharmacist_3*
**Understanding that PROMPPT is not a one-off consultation but a longer-term process with continued support for patients**	'Yeah, it's not just about these 20 to 30 minutes today, it's about a much bigger, longer term picture' *IPT pharmacist_3*<br><br>'I think that's a thing to bear in mind it's not everything has to be done in one consultation, you can approach it at the next one and I think that was where the conversation got today eventually - people think we have to fit everything in one' *IPT pharmacist_2*
**OPPORTUNITY COSTS**	
**PROMPPT needs to fit with the patients' priorities to enable engagement**	'I think it's important that it fits with their life at that time. Because again, there could be things that are going on that's going to impact the outcomes really. Because again, if somebody is going through a hard time emotionally or anything else, the chances are that it's gonna come down quite low on their list of changes they're gonna probably wanna make' *IPT pharmacist_3*
**PERCEIVED EFFECTIVENESS**	
**PROMPPT was effective in achieving its aims**	'I think it went okay cause I was clear that actually we were trying to take away the co-codamol, I'd already sort of said to him, he was on a minimum amount of analgesia, erm, but it was worth trying to drop it down a little bit if he could, cause he was very much, well I get back pain during gardening then what, I was like, well obviously you still have your co-codamol there, he was like, right okay, so that's, it sort of worked, it was quite subtle and it was quite quick, that moment but I think it gave enough reassurance for him to go, okay that's fine' *IPT pharmacist_2*<br><br>'I suppose we gave him a few ideas about setting seeds for the future in terms of trying to lay foundations of, lets try and reduce your analgesia down as much as we can just in case we get a flare, erm up especially with his IBD, as well erm, condition but he did the hard work for me…we've posed ideas and we've thought about things for the future and we've put in erm, we've

	suggested actually if his pain escalates, come back in so erm, so yeah, there wasn't anything that was outright, this is a massive achievement' *IPT pharmacist_2*<br><br>'Again it doesn't mean it's not a worthwhile conversation because perhaps it gives the patient a bit more mindfulness about going forward with a chronic pain condition, about reducing the risk of escalation and doses I suppose as well. So maybe there's more benefit just around that sort of idea, rather than reducing today' *IPT pharmacist_3*
**Optimistic that PROMPPT will be successful in the long-term**	'Hopefully a long term erm reduction in pain – opioid pain relief use' *IPT pharmacist_1*<br><br>'It's just got more – it's a stronger opinion erm because actually look how beneficial it's been. Not just with – probably with all the patients that we've seen, they've all benefitted from it' *IPT pharmacist_2*<br><br>'I think it can – so to reduce the burden of opioid use. So long-term risks, reduce mental health problems, erm I mean ultimately unnecessary deaths as well from excessive use. So yeah, I think there's a big scope, big potential. And there's also a cost-saving element as well isn't there, obviously. I mean outside of all that it's the use of more and more is gonna cost more and more, so' *IPT pharmacist_3*
**Patients will see value in the review and will also feel supported and valued as a result**	'Patients having greater confidence in the practice pharmacist and sort of feeling listened to' *IPT pharmacist_1*<br><br>'So I think maybe it's a reassurance that – ongoing that she's actually doing something – that she's reassuring herself that she's doing something about it in between. That it's not just left between her consultant appointments with no kind of review. So maybe – and again, giving her that opportunity just to you know, reassure that she's – or to tell – cos it could be that she just want to tell us that she has reduced. So that she's not taking eight a day, so actually she's informing us that she is taking less and she is responsible about taking them. And again, and so maybe that could've been a beneficial part of today for her' *IPT pharmacist_3*<br><br>'The action plan you know, there should be some sort of action at the end, rather than just, 'Thank you for coming' and that's everything done. I think there needs to be something that's kind of followed through from it. And that's a bit of a pressure again in terms of knowing you're kind of building towards something, however small it might seem, just to give the patient that reassurance that they've come away from it with something more than they had when they came in. Because they can, that ultimately in itself is probably detrimental to their ongoing management isn't it then? You need to kind of at least give them some – and there's that hope that they're being managed better in some way by – from the contact...Yeah, value. And whether that's you know – it doesn't have to be necessarily an improvement in their pain relief at that time. But at least it could be – like you say, well as we're trying to do, the risk reduction over a period of time. So it might just be something that they just reflect

	on later and say, 'Well yeah, somebody's at least trying, they're looking out for you' basically' *IPT pharmacist_3*
**Patient resistance will affect tapering success**	'His barriers are up and there's just no – you know, even sometimes tapping away at them to see if they're interested in other options, but it was like he wasn't really' *IPT pharmacist_2*
**The structure and review components allowed the consultations to flow, making it effective**	'Erm, I feel the consultation went really well, I mean in terms of it just flowed didn't it, erm, but I think it was just set up, the agenda was known, so actually there was no locking of horns so he knew why he was here, he knew why we were here, try to make it very clear that it was an open conversation rather than a purposeful, we're going to do X, Y and Z and then it just allowed it to follow its pattern' *IPT pharmacist_2*<br><br>'I think it was really quite simple. Cos again, because things like the resources were all there, the patients brought in the pains concern form, so you're not having to illicit all the information yourself. As I say, yeah it was – yeah, as consultations go it was fairly – just asking the question and it's about the patients. There's no observations or you know, no obvs you have to take or anything. So as a consultation, yeah it's fairly – yeah it was fairly easy to be honest, for that reason' *IPT pharmacist_3*
**Pharmacists felt they could or should have done more to make a difference**	'I suppose when I get to the end of it and realise that I've [laughs] – cos my head as a pharmacist is like 'I need to change something on the medication front' and I got to the end of it and thought, 'I've actually done nothing with the medication'. So I suppose – not that I'd said wholeheartedly disliked it, but I felt a bit dissatisfied that, 'Have I really made a difference there?'' *IPT pharmacist_1*<br><br>'Not being able to make a difference…Not having that skill to try and influence and change his mind-set and his beliefs. Which I know is not necessarily – I can't just you know, I can't expect that in every consultation I have, but yeah, it's just really hard' *IPT pharmacist_2*<br><br>'So again, he's on – been on a slightly lower grade of dose. So you know, just two twice a day. It sometimes perhaps feels like you're not being kind of – not making a significant enough change at that point. But I think it's just being kind of mindful that you know, any change might well be quite a big change to them' *IPT pharmacist_3*
**SELF-EFFICACY**	
**Prior experience allows pharmacists to feel confident to deliver PROMMPT**	'Erm, this one went really well….but then I've got quite a bit of experience to back me up, does that make sense? But then I still worry' *IPT pharmacist_2*<br><br>'I didn't feel uncomfortable about asking about her usage. It's something I do every day with medication. So I think that would go the same, whether it was somebody on high doses of morphine or just on occasional Tramacet like she was on. So not an

	issue' *IPT pharmacist_1*<br><br>'I think personally I do my prescribing in pain so I learnt how to prescribe, the course we have to do as Pharmacists, I did my area of expertise was pain, so I'm a bit privileged in terms of this study' *IPT pharmacist_2*
**Resistant patients can knock pharmacist's confidence**	'So my confidence sort of just [laughs] took a bit of a knock halfway through. Well, less than halfway through sadly [laughs]' *IPT pharmacist_2*<br><br>'It just knocked my confidence [laughs]. Only slightly, but you know, you win some and you lose some. And I know – I do recognise that, you know. I've got people on far higher doses of Fentanyl. Erm, I've had a similar conversation with somebody in that respect and it was the same thing. I've been there before, it's a brick wall. 'It works for me, that's what I need and whatever you're saying to me is not going to help me because I know myself'. So I've been there before. So it wasn't – you know, it wasn't that much of a shock cos I know that there's people like that. But it's just disappointing isn't that you can't make a difference' *IPT pharmacist_2*
**Consultations skills are more important than having the clinical skills to deliver PROMPPT**	'Actually it's not about the medical training, which pharmacists probably we're more worried and cautious about, it's actually about the consultation skills and the rapport. Does that make sense? So it is really focusing on that and giving them the confidence to be able to do it, to get over that difficulty of going, 'Oh no, I'm not doing it'. Does that make sense?' *IPT pharmacist_2*
**Pharmacists not as confident in discussing self-care and non-pharmacological alternatives**	'It's harder to talk about self-management than it is to talk about analgesia, if we're all honest about it. It's the way we're taught isn't it' *IPT pharmacist_2*<br><br>'Erm, so a better understanding of the physio options. And I think – I remember when I shadowed at the pain clinic - and she was talking to a patient about looking at people's activities at home and modifying things for them to help them manage better. So maybe again, having a bit more insight as to what those sort of things are, so then you can just you know, tell the patient about it a bit more and say, 'Would you be interested in exploring this further?'' *IPT pharmacist_1*
**Generally confident to make referrals but more knowledge on available services**	'She's got no faith in it whatsoever and I didn't push that. Probably due to my lack of knowledge about what physio can actually bring to those sort of you know, lower back pain. I admittedly never really sort of looked into what physio can bring to that pain or – well, it's not pain is it, it's improving the activity. But yeah, I can see that she wasn't – she wasn't really interested in it. She's doing her swimming, she's doing – she's keeping active. So I wouldn't have necessarily explored it any further. But I think maybe as a pharmacist, if I'd got a bit more insight as to the options for physio, it might help me in terms of trying to sell it to them a bit

**would help**	better' *IPT pharmacist_1*<br><br>'Erm, I think it's the physio side of it that I probably just need to expand the knowledge a bit more. Maybe we need some sort of background on how it works a bit more' *IPT pharmacist_1*
